# An Efficient Three Component One-Pot Synthesis of 5-Amino-7-aryl-7,8-dihydro-[1,2,4] triazolo[4,3-a]-pyrimidine-6-carbonitriles

**DOI:** 10.3390/molecules17021860

**Published:** 2012-02-14

**Authors:** Keyume Ablajan, Wetengul Kamil, Anagu Tuoheti, Sun Wan-Fu

**Affiliations:** College of Chemistry and Chemical Engineering, Xinjiang University, Urumqi 830046, China

**Keywords:** 1,2,4-triazolo[4,3-a]pyrimidine, three component reactions, one-pot synthesis, ultrasound irradiation

## Abstract

A series of novel 5-amino-7-aryl-7,8-dihydro-[1,2,4] triazolo[4,3-a]-pyrimidine-6-carbonitriles were synthesized by a one-pot reaction of 3-amino-1,2,4-triazole, malononitrile and aryl aldehydes in the presence of 20 mol% NaOH in ethanol under heating or ultrasonic irradiation. The structures of the target compounds were confirmed by inspection of their ^1^H- NMR, ^13^C-NMR, IR and MS spectra. The advantages of this method are short reaction times, good yields, high selectivity and operational simplicity.

## 1. Introduction

Heterocyclic compounds have drawn special attention in organic chemistry because of their abundance in natural products and their diverse biological properties [1]. Pyrimidine and its derivatives have been recognized as important heterocyclic compounds due to their variety of chemical and biological significance to medicinal chemistry [2,3,4]. It is well known that the condensation of aminoheterocycles and pyrimidine gives rise to the formation of bicyclic heterocycles known as triazolopyrimidines [5]. The 1,2,4-triazolopyrimidines have attracted growing interest due to their important pharmaceutical properties [6,7,8], and they appear in a variety of synthetic pharmacophores which possess anti-parasitic, antimicrobial, anticancer and antibiotic activities [9,10,11].

Multi-component reactions (MCRs), by virtue of their flexibility to rapidly assemble three or more reactants and convert them into higher molecular weight compounds in one-pot [12], have become very popular in the discovery of biologically active novel compounds due to their experimental simplicity, atom economy and high product yields [13]. The one pot synthesis of multisubstituted 1,4-dihydropyridines through multi-component reactions was reported previously [14,15,16]. Three-component one-pot pathways were also used to synthesize hydroxylated triazolopyrimidines and multisubstituted multisubstituted pyrimido[1,2-b]benzimidazole derivatives [9,17]. In addition, the syntheses of some fused triazolo- pyrimidines derivatives using multicomponent one pot methods were also reported [18].

Ultrasonic irradiation is a powerful tool in organic reactions due to its advantages such as convenient operation, mild conditions and higher yields [19]. Ultrasound has been employed in different kind of organic synthesis and has become popular for constructing new heterocycles [20,21,22].

## 2. Results and Discussion

### 2.1. Synthesis

**Scheme 1:**
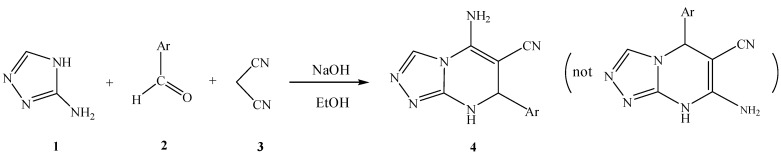
The one pot synthesis of multi-substituted triazolo[4,3-a]pyrimidine derivatives under ultrasound irradiation.

In a continuation of our interest on the synthesis new heterocyles using multicomponent one-pot methods, herein we report the one-pot synthesis of 5-amino-7-aryl-7,8-dihydro-[1,2,4]-triazolo[4,3-a] pyrimidine-6-carbonitriles via three component reactions of 3-amino-1,2,4-triazole, aromatic aldehydes and malononitrile in the presence of NaOH under heating or ultrasonic irradiation at room temperature. The one-pot reaction is shown in Scheme 1. Interestingly, triazolo[4,3-a]pyrimidines **4** were obtained in moderate to good yields using 20 mol% NaOH as a catalyst under both heating and reflux conditions. The similar reaction under microwaves or ultrasonic waves was reported previously [23], however, the final products were different from those reported in our current paper (Scheme 1).

To determine the optimal reaction conditions, the one pot reactions between 3-amino-1,2,4-triazole, malononitrile and 4-chlorobenzaldehyde (**3h**) in the presence of different catalysts and in different solvents via heating at 80 °C for 30 min, respectively, were examined (Table 1). The results show that the final product **4h** was not formed (entry 2**-**4) when the NaOH was replaced by Et_3_N, L-proline or HOAc. Thus, NaOH was chosen as an optimal catalyst for further investigation. Furthermore, the same reaction was carried out using different solvents in the presence of NaOH (20% mol) as a catalyst, respectively. As shown in Table 1, the desired product **4h** was formed in low yields (30%, 35% and 40%, respectively) when H_2_O, CH_2_Cl_2_ and MeCN was chosen as solvent. In order to confirm the best method for the synthesis of multisubstituted triazolo[4,3-a]pyrimidine derivatives, the reaction was also carried out using ultrasonic waves as an assisted technique.

**Table 1:** Optimization of reaction conditions for multi-substituted triazolo[4,3-a]pyrimidine (**4h**).

Entry	Catalyst	Amount (% mol)	Solvent	Method A ^a^			Method B ^b^	
				Time (min)	Yields (%) ^c^		Time (min)	Yields (%) ^c^
1	―	―	EtOH	30	―		60	―
2	Et_3_N	20	EtOH	30	―		60	―
3	L-proline	20	EtOH	30	―		60	―
4	HOAc	20	EtOH	30	―		60	―
5	NaOH	20	EtOH	30	85		60	88
6	NaOH	20	H_2_O	30	30		60	35
7	NaOH	20	CH_2_Cl_2_	30	35		60	40
8	NaOH	20	CH_3_CN	30	40		60	45
9	NaOH	5	EtOH	30	50		60	54
10	NaOH	10	EtOH	30	60		60	63
11	NaOH	15	EtOH	30	75		60	78
12	NaOH	30	EtOH	30	85		60	88
13	NaOH	50	EtOH	30	85		60	88

^a^ Reaction under reflux conditions; ^b^ Reaction under ultrasonic waves at 25~30 °C and ultrasonic power 250 W, irradiation frequency 25 Khz; ^c^ Isolated yields.

The results obtained from sonication were summarized in Table 1. As seen in Table 1, the reaction performed under sonication afforded comparatively higher yields within 60 min without formation of any side-products. The efficiency and promoting effect of ultrasound to reaction system was discussed in detail previously [24,25]. According to the method and procedure described in literature and optimization of irradiation frequency, an optimal frequency of 25 kHz was set at for all reactions.

In addition, we attempted the reactions to test the optimal amount of base catalyst under both heating and ultrasound irradiation conditions. When the catalytic amount of NaOH used for this reaction were 5%, 10%, 15% and 20%, respectively, the yields of product gradually increased within 30 min and 60 min for both methods. Interestingly, the reactions afforded the product **4h** in almost the same yield (88%) when using 20 mol%, 30 mol% and 50 mol% NaOH within 60 min under ultrasonic irradiation. Therefore, it can be summarized that the reaction give a good yield under optimized reaction conditions involving the use of 20 mol% NaOH as a catalyst in EtOH solvent.

Based on the optimized reaction conditions established above, a series of novel 5-amino-7-aryl-7,8-dihydro-[1,2,4]-triazolo[4,3-a]pyrimidine-6-carbonitriles were next prepared under catalysis of NaOH involving different aldehydes (Table 2). All reactions were completed within 60 min under ultrasonic irradiation. The results show that aromatic aldehydes with electron-withdrawing and electron donating groups, as well as heteroaromatic aldehydes, can be successfully reacted with 3-amino-1,2,4-triazole and malononitrile to give the desired productd **4a–4m** in moderate to good yields.

**Table 2:** The synthesis of multisubstitued [1,2,4]-triazolo[4,3-a] pyrimidines under heating and ultrasonic irradiation.

Entry	Ar	Product	Method A ^a^		Method B ^b^
			Time(min)/Yields (%) ^c^		Time(min)/Yields (%) ^c^
1	4-Me-C_6_H_4_	**4a**	30/70		60/75
2	4-MeO-C_6_H_4_	**4b**	30/74		60/80
3	3,4-2MeO-C_6_H_4_	**4c**	30/78		60/84
4	3-HO-C_6_H_4_	**4d**	30/78		60/84
5	4-F-C_6_H_4_	**4e**	30/80		60/86
6	4-Br-C_6_H_4_	**4f**	30/85		60/90
7	2-Cl-C_6_H_4_	**4g**	30/70		60/75
8	4-Cl-C_6_H_4_	**4h**	30/82		60/88
9	2,4-2Cl-C_6_H_4_	**4i**	30/78		60/83
10	4-O_2_N-C_6_H_4_	**4j**	30/60		60/67
11	4-Me_2_N-C_6_H_4_	**4k**	30/79		60/84
12	3-Pyridyl	**4l**	30/75		60/80
13	4-Pyridyl	**4m**	30/68		60/75

^a^ Reaction under reflux conditions; ^b^ Reaction under ultrasonic waves at 25~30 °C and ultrasonic power 250 W, irradiation frequency 25 Khz; ^c^ Isolated yields.

**Scheme 2:**
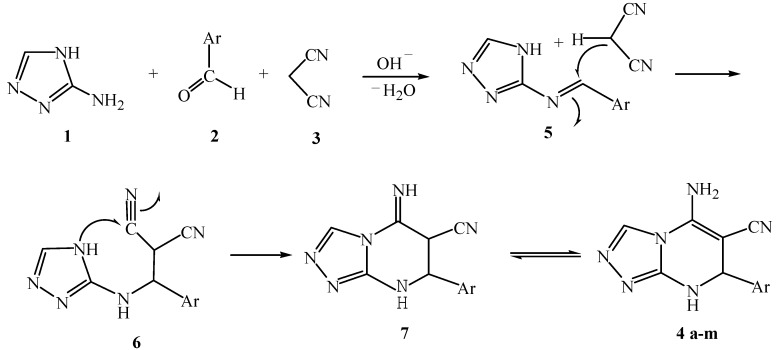
Proposed mechanism for the formation of [1,2,4]-triazolo [4,3-a]-pyrimidines.

The mechanism of the three-component, one-pot reaction was assumed to be as follows: at first condensation occurred between aminotriazole **1** and aldehyde **2** to afford an intermediate **5**, and then the subsequent reaction between intermediate **5** and malononitrile (**3**) takes place to form the intermediate **6**. Furthermore, the intramolecular Michael addition between the NH and CN groups of the intermediate molecule **6** resulted in the formation of an intermediate product **7**, which was further converted to a final product **4** through isomerization (Scheme 2).

### 2.2. Spectral Analysis

The structures of products were confirmed on the basis of their spectral data. The IR spectra of 7,8-dihydro-5-amino-6-cyano-7-aryl-[1,2,4]-triazolo[4,3-a] pyrimidines **4a–m** show characteristic absorption bands of a conjugated CN group at 2198–2182 cm^−1^, and broad absorption bands of NH and NH_2_ at 3427–3111 cm^−1^. In the ^1^H-NMR spectra, the NH_2_ signal was found at 6.69–7.36 ppm as a singlet, and the aromatic protons was found at 6.70–8.60 ppm as multiplets. A doublet found at 8.69–8.93 ppm corresponded to a NH proton in the pyrimidine ring, as determined by deuterium exchange tests. As a result, a doublet observed at 5.18–5.77 ppm that was assigned to a CH proton in the pyrimidine rings changed to a singlet. Therefore, the coupling (*J* = 2.3 Hz) between the NH proton and the CH proton located in the pyrimidine ring and their correlation of them could be determined. The spectra for product **4j** are given in the Supporting Information. Additionally, HMBC experiments was performed to determine the structures of products. For instance, the structure of **4a** was confirmed according to the appearance of a cross peak between proton and carbon atoms obtained by such HMBC experiments.

## 3. Experimental

### 3.1. General

The melting points were recorded on a Buchi B2540 microscopic melting apparatus. Infrared (IR) spectra were obtained on a Bruker-Equinox 55 spectrophotometer in KBr pellets. The ^1^H-NMR spectra were measured on a Varian Inova 400 NMR spectrometer in DMSO-*d*_6_ solutions using TMS as internal standard. *J* values are in Hz. Chemical shifts (δ) were reported in parts per million (ppm). Mass spectra were measured with an API 2000 spectrometer. Sonication was performed in an ultrasound cleaner with a frequency of 25 kHz through manual adjustment and an output power of 250 W. The reaction mixture in flask was placed at the centre of the cleaner. The temperature of the water bath was controlled at 25~30 °C. All chemicals were purchased from commercial sources and used without further purification. Analytical samples were dried at room temperature.

### 3.2. General Procedure for the Synthesis of Multisubstituted Triazolo[4,3-a]pyrimidines ***4***

*Method A*: To a mixture of an 3-amino-1,2,4-triazole (1 mmol), malononitrile (1 mmol) and aromatic aldehyde (1 mmol) in ethanol (10 mL) was added the catalyst NaOH (20% mmol) and the reaction mixture was refluxed for 1–2 h. After completion of the reaction as indicated by TLC, the reaction mixture was cooled, filtrated and the solid residue was washed with ethanol (10 mL). The corresponding product **4** was obtained in high purity after crystallization of the crude product from ethanol.

*Method B*: A mixture of an 3-amino-1,2,4-triazole (1 mmol), malononitrile (1 mmol), aromatic aldehyde (1mmol) and NaOH (20% mmol) in ethanol (10 mL) was sonicated in the water bath of an ultrasonic cleaner under air at 25~30 °C for 60 min (monitored by TLC). The resulting crude products were isolated and washed with ethanol (10 mL). The corresponding pure products **4** were obtained by crystallization from ethanol.

*5-Amino-7-(4-Methylphenyl)-7,8-dihydro-[1,2,4]-triazolo[4,3-a]pyrimidine-6-carbonitrile* (**4a**). White tiny crystals; mp = 245−246 °C. ^1^H-NMR *δ*: 2.29 (s, 3H, CH_3_), 5.28 (d, 1H, *J* = 2.4 Hz, CH), 7.15 (s, 2H, NH_2_), 7.18−7.19 (dd, 4H, *J* = 8.4 Hz, *p*-H_3_C-C_6_H_4_), 7.70 (s, 1H, CH), 8.73 (d, 1H, *J* = 2.4 Hz, NH). ^13^C-NMR *δ*: 20.57, 53.64, 56.02, 118.97, 125.92 (2C), 129.10 (2C), 137.17, 140.19, 146.85, 151.75, 153.86. IR (υ/cm^−1^): 3241, 3182, 3115, 3046, 2193, 1661, 1631, 1531, 1482, 1449, 1213, 1154. ESI-MS, m/z: 253 [M+H]^+^, 275 [M+Na]^+^.

*5-Amino-7-(4-methoxyphenyl)-7,8-dihydro-[1,2,4]-triazolo[4,3-a]pyrimidine-6-carbonitrile* (**4b**). White tiny crystals; mp = 218−219 °C. ^1^H-NMR *δ*: 3.74 (s, 3H, OCH_3_), 5.27 (s, 1H, *J* = 2.4 Hz, CH), 6.93 (d, 2H, *J* = 8.4 Hz, *p*-H_3_CO-C_6_H_4_), 7.22 (d, 2H, *J* = 8.4 Hz, *p*-H_3_CO-C_6_H_4_), 7.17 (s, 2H, NH_2_), 7.69 (s, 1H, CH), 8.70 (s, 1H, NH). ^13^C-NMR *δ*: 53.39, 55.11, 56.18, 113.90 (2C), 118.96, 127.34 (2C), 135.12, 146.81, 153.80, 158.92. IR (υ/cm^−1^): 3368, 3263, 3134, 3030, 2184, 1683, 1628, 1513, 1481, 1251, 1178, 1025. ESI-MS, m/z (%): 269 [M+H]^+^, 291 [M+Na]^+^. 

*5-Amino-7-(3,4-dimethoxyphenyl)-7,8-dihydro-[1,2,4]-triazolo[4,3-a]pyrimidine-6-carbonitrile* (**4c**). White crystal; mp = 235−237 °C. ^1^H-NMR *δ*: 3.73 (s, 3H, OCH_3_), 3.74 (s, 3H, OCH_3_), 5.28 (d, 1H, *J* = 2.4 Hz, CH), 6.78−6.80 (dd, 1H, *J* = 2.4 Hz, *J* = 8.0 Hz, H_6'_), 6.93 (d, 1H, *J* = 2.0 Hz, H_2'_), 6.94 (d, 1H, *J* = 8.4 Hz, H_5'_), 7.18 (s, 2H, NH_2_), 7.70 (s, 1H, CH, H_6_), 8.69 (d, 1H, *J* = 2.4 Hz, NH). ^13^C-NMR *δ*: 53.63, 55.36, 55.43, 55.96, 110.11, 111.57, 118.13, 118.98, 135.25, 146.93, 148.51, 148.63, 151.72, 153.83. IR (υ/cm^−1^): 3409, 3243, 3161, 3099, 2198, 1685, 1622, 1519, 1478, 1240, 1141, 1019. ESI-MS, m/z: 299 [M+H]^+^, 321 [M+Na]^+^.

*5-Amino-7-(3-hydroxylphenyl)-7,8-dihydro-[1,2,4]-triazolo[4,3-a]pyrimidine-6-carbonitrile* (**4d**). White powder; mp = 251−253 °C. ^1^H-NMR *δ*: 5.21 (d, 1H, *J* = 2.4 Hz, CH), 6.69 (s, 2H, NH_2_), 6.70−7.37 (m, 4H, *m*-HO-C_6_H_4_), 7.71 (s, 1H, CH), 8.75 (d, 1H, *J* = 2.4 Hz, NH). IR (υ/cm^−1^): 3384, 3312, 3129, 2177, 1648, 1587, 1514, 1495, 1354, 1218, 1131. ESI-MS, m/z: 255 [M+H]^+^, 277 [M+Na]^+^.

*5-Amino-7-(4-fluorophenyl)-7,8-dihydro-[1,2,4]-triazolo[4,3-a]pyrimidine-6-carbonitrile* (**4e**). White tiny crystals; mp = 252−254 °C. ^1^H-NMR *δ*: 5.38 (d, 1H, *J* = 2.4 Hz, CH), 7.20 (s, 2H, NH_2_), 7.22−7.35 (dd, 4H,*J* = 8.8 Hz,*p*-F-C_6_H_4_), 7.71 (s, 1H, CH), 8.78 (d, 1H, *J* = 2.0 Hz, NH). IR (υ/cm^−1^): 3237, 3183, 3116, 2194, 1658, 1599, 1529, 1483, 1360, 1220, 1157. ESI-MS, m/z: 257 [M+H]^+^, 279 [M+Na]^+^.

*5-Amino-7-(4-bromolphenyl)-7,8-dihydro-[1,2,4]-triazolo[4,3-a]pyrimidine-6-carbonitrile* (**4f**). White tiny crystal; mp = 264−266 °C. ^1^H-NMR *δ*: 5.37 (d, 1H, *J* = 2.0 Hz, CH), 7.25 (s, 2H, NH_2_), 7.26 (d, 2H,*J* = 8.4 Hz,*p*-Br-C_6_H_4_), 7.60 (d, 2H,*J* = 8.4 Hz,*p*-Br-C_6_H_4_), 7.72 (s, 1H, CH), 8.81 (d, 1H, *J* = 2.4 Hz, NH). ^13^C-NMR *δ*: 53.28, 55.43, 118.80, 121.04, 128.32 (2C), 131.53 (2C), 142.40, 146.98, 151.84, 153.76; IR (υ/cm^−1^): 3238, 3179, 3119, 2916, 2195, 1658, 1631, 1533, 1482, 1355, 1152, 1070. ESI-MS, m/z: 317 [M+H]^+^, 339 [M+Na]^+^.

*5-Amino-7-(2-chlorophenyl)-7,8-dihydro-[1,2,4]-triazolo[4,3-a]pyrimidine-6-carbonitrile* (**4g**). White crystal; mp = 263−266 °C. ^1^H-NMR *δ*: 5.76 (s, 1H, CH), 7.26 (s, 2H, NH_2_), 7.35−7.49 (m, 4H, *O*-Cl-C_6_H_4_), 7.71 (s, 1H, CH), 8.76 (s, 1H, NH). ^13^C-NMR *δ*: 51.98, 54.80, 118.33, 127.78, 129.06, 129.82, 131.43, 139.47, 147.15, 151.75, 153.76. IR (υ/cm^−1^): 3357, 3307, 3162, 3128, 2182, 1660, 1631, 1521, 1480, 1368, 1150; ESI-MS, m/z: 273 [M+H]^+^, 295 [M+Na]^+^.

*5-Amino-7-(4-chlorophenyl)-7,8-dihydro-[1,2,4]-triazolo[4,3-a]pyrimidine-6-carbonitrile* (**4h**). White crystals; mp = 257−258 °C. ^1^H-NMR *δ*: 5.39 (d, 1H, *J* = 2.4 Hz, CH), 7.27 (s, 2H, NH_2_), 7.33 (d, 2H, *J* = 8.8 Hz, *p*-Cl-C_6_H_4_), 7.46 (d, 2H, *J* = 8.8 Hz, *p*-Cl-C_6_H_4_), 7.72 (d, 1H, CH), 8.81 (d, 1H, *J* = 2.0 Hz, NH). ^13^C-NMR *δ*: 53.22, 55.49, 118.83, 127.98 (2C), 128.62 (2C), 132.49, 141.99, 146.99, 151.84, 153.77. IR (υ/cm^−1^): 3497, 3234, 3181, 3116, 2918, 2196, 1654, 1631, 1594, 1533, 1483, 1356, 1090. ESI-MS, m/z: 273 [M+H]^+^, 295 [M+Na]^+^.

*5-Amino-7-(2,4-dichlorophenyl)-7,8-dihydro-[1,2,4]-triazolo[4,3-a]pyrimidine-6-carbonitrile* (**4i**). White powder; mp = 274−277 °C. ^1^H-NMR *δ*: 5.77 (d, 1H, *J* = 2.0 Hz, CH), 7.32 (s, 2H, NH_2_), 7.43−7.45 (d, 1H, *J* = 8.0 Hz, H_6'_), 7.48−7.51 (dd, 1H, *J* = 8.4 Hz, *J* = 2.0 Hz, H_5'_), 7.66 (d, 1H, *J* = 2.0 Hz, H_3'_), 7.72 (s, 1H, CH), 8.68 (d, 1H, *J* = 1.6 Hz, NH). ^13^C-NMR *δ*: 51.64, 54.43, 118.20, 127.97, 129.20, 130.59, 133.00, 133.42, 138.51, 147.19, 151.78, 153.69. IR (υ/cm^−1^): 3363, 3254, 3161, 2191, 1631, 1522, 1482, 1460, 1361, 1021, 1099. ESI-MS, m/z: 308 [M+H]^+^, 330 [M+Na]^+^.

*5-Amino-7-(4-nitrophenyl)-7,8-dihydro-[1,2,4]-triazolo[4,3-a]pyrimidine-6-carbonitrile* (**4j**). Brown powder; mp = 245−247 °C. ^1^H-NMR *δ*: 5.55 (d, 1H, CH), 7.34 (s, 2H, NH_2_), 7.55 (d, 2H, *J* = 8.8 Hz, *p*-O_2_N-C_6_H_4_), 7.73 (d, 1H, CH), 8.25 (d, 2H, *J* = 8.2 Hz, *p*-O_2_N-C_6_H_4_), 8.92 (d, 1H, *J* = 2.0 Hz, NH). ^13^C-NMR *δ*: 53.15, 54.79, 118.68, 124.02 (2C), 127.30 (2C), 147.06, 147.15, 150.11, 151.96, 153.75. IR (υ/cm^−1^): 3427, 3247, 3078, 2188, 1692, 1626, 1518, 1485, 1349, 1153. ESI-MS, m/z: 284 [M+H]^+^, 306 [M+Na]^+^.

*5-Amino-7-(4-N,N-dimethylaminophenyl)-7,8-dihydro-[1,2,4]-triazolo[4,3-a]pyrimidine-6-carbonitrile* (**4k**). Yellow powder; mp = 242−245 °C. ^1^H-NMR *δ*: 5.18 (d, 2H, *J* = 2.0 Hz, CH), 6.70 (d, 2H, *J* = 8.8 Hz, *p*-(H_3_C)_2_N-C_6_H_4_), 7.09 (d, 2H, *J* = 8.8 Hz,*p*-(H_3_C)_2_N-C_6_H_4_), 7.12 (s, 2H, NH_2_), 7.68 (s, 1H, CH), 8.62 (d, 1H, *J* = 2.4 Hz, NH). ^13^C-NMR *δ*: 53.54, 56.46, 68.51 (2C), 111.72 (2C), 112.16, 115.44, 118.64, 126.83, 133.50 (2C), 150.129, 154.24, 158.78. IR (υ/cm^−1^): 3332, 3232, 3118, 2188, 1690, 1621, 1555, 1521, 1490, 1372, 1155. ESI-MS, m/z: 282 [M+H]^+^, 304 [M+Na]^+^.

*5-Amino-7-(3-pyridyl)-7,8-dihydro-[1,2,4]-triazolo[4,3-a]pyrimidine-6-carbonitrile* (**4l**). White powder; mp = 240−242 °C. ^1^H-NMR *δ*: 5.48 (d, 1H, *J* = 2.0 Hz, CH), 7.33 (s, 2H, NH_2_), 7.42−7.45 (q, 1H,* J* = 8.0 Hz, *J* = 4.8 Hz, pyridyl-H), 7.70−7.73 (dd, 1H, *J* = 8.0 Hz, *J* = 2.0 Hz, pyridyl-H), 7.74 (s, 1H, CH), 8.53 (d, 1H, *J* = 2.4 Hz, pyridyl-H), 8.53−8.55 (dd, 1H, *J* = 2.0 Hz, *J* = 5.2 Hz, pyridyl-H), 8.82 (d, 1H, *J* = 2.0 Hz, NH). IR (υ/cm^−1^): 3254, 3109, 2190, 1685, 1637, 1578, 1518, 1489, 1370, 1152. ESI-MS, m/z: 240 [M+H]^+^, 262 [M+Na]^+^.

*5-Amino-7-(4-pyridyl)-7,8-dihydro-[1,2,4]-triazolo[4,3-a]pyrimidine-6-carbonitrile* (**4m**). Brick colored powder; mp = 250−252 °C. ^1^H-NMR *δ*: 5.42 (d, 1H, *J* = 2.4 Hz, CH, H_4_), 7.28−7.297 (dd, 2H, *J* = 4.4 Hz, pyridyl-H), 7.36 (s, 2H, NH_2_), 7.75 (s, 1H, CH), 8.58−8.597 (dd, 2H, *J* = 4.4 Hz, pyridyl-H), 8.93 (d, 1H, *J* = 2.4 Hz, NH). ^13^C-NMR *δ*: 52.66, 54.42, 118.75, 120.80 (2C), 147.28, 150.06 (2C), 151.29, 151.95, 153.92. IR (υ/cm^−1^): 3325, 3228, 3111, 2190, 1676, 1643, 1557, 1515, 1490, 1360, 1148. ESI-MS, m/z: 240 [M+H]^+^, 262 [M+Na]^+^.

## 4. Conclusions

We have developed a concise and a convenient new strategy for the synthesis of novel multi- substituted triazolo[4,3-a]pyrimidine derivatives in high yields from 3-amino-1,2,4-triazole, malononitrile and aryl aldehydes using 20 mol% NaOH as a catalyst. This method is mild and convenient for the preparation of multisubstituted triazolopyrimidines in a one-pot operation.

## Supplementary Materials

Supplementary materials can be accessed at: http://www.mdpi.com/1420-3049/17/2/1860/s1.
