# Supplementary material for: An Efficient Three Component One-Pot Synthesis of 5-Amino-7-aryl-7,8-dihydro-[1,2,4] triazolo[4,3-a]-pyrimidine-6-carbonitriles
Source: Molecules. 2012 Feb 14;17(2):1860–9. doi: 10.3390/molecules17021860 (PMC6268426; doi:10.3390/molecules17021860)
Supplement: Supplementary file 1 [file molecules-17-01860-s001.pdf]

Supplementary Materials

Figure S1: The D_2O exchange experiment for product **4j**. (**A**) Partial enlarged ^{1}H-NMR spectrum of **4j** in DMSO-d_6; (**B**) Partial enlarged ^{1}H-NMR spectrum of **4j** in DMSO-d_6 + $\mathbf{D_2O}$.

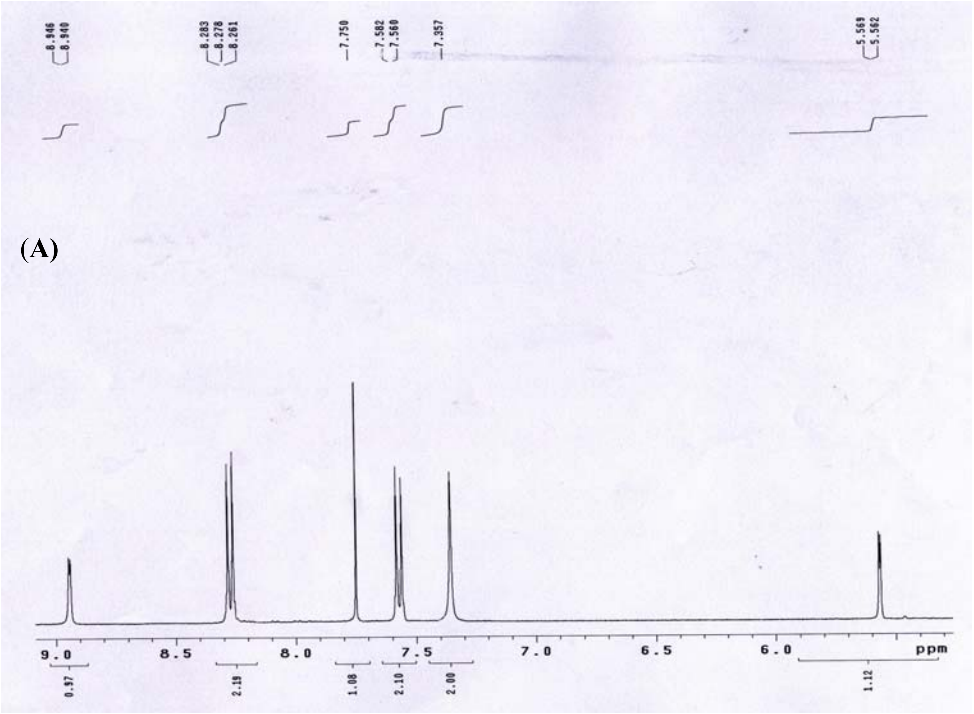

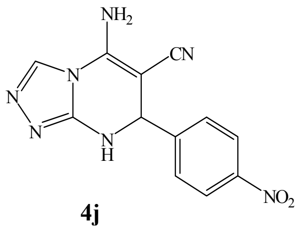

4j

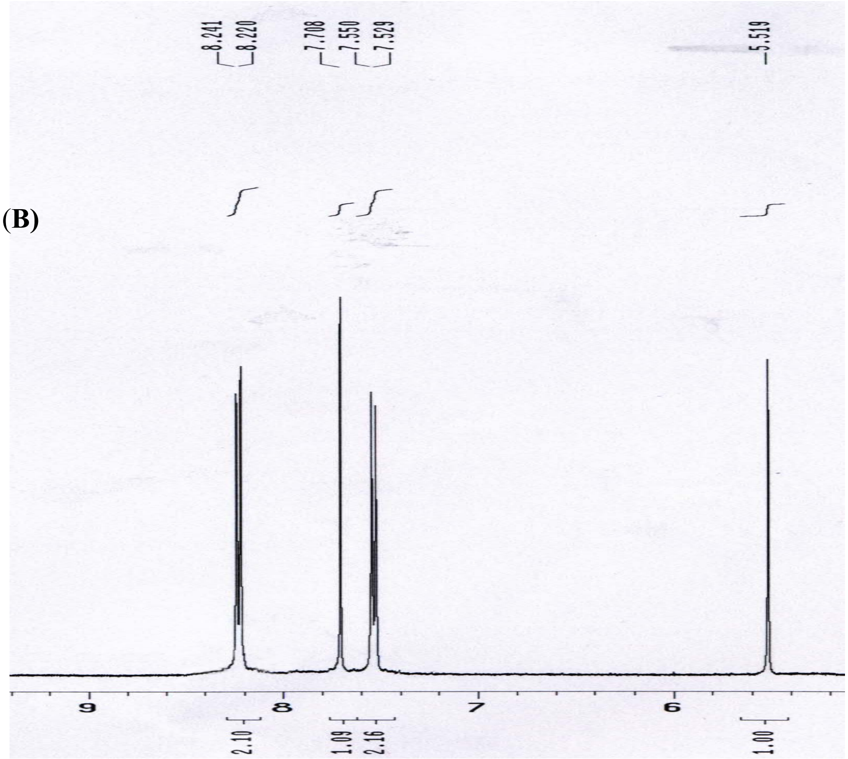

Figure S2. (A) The ^{13}C NMR spectrum of compound 4a in DMSO; (B) The DEPT ^{13}C NMR spectrum of compound 4a in DMSO.

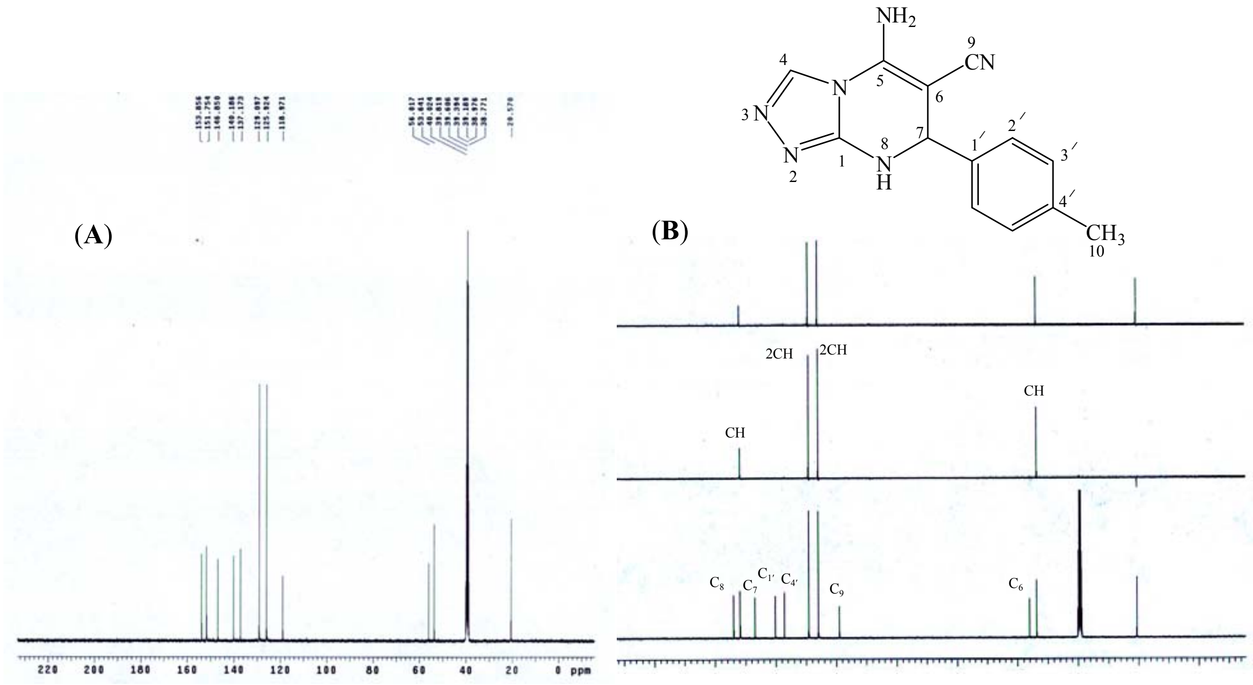

Figure S3. (**A**) The 2-D ^{1}H-^{13}C gHMBC NMR spectrum of compound **4a** in DMSO; (**B**) HMBC correlation with ^{1}H and ^{13}C NMR of **4a**.

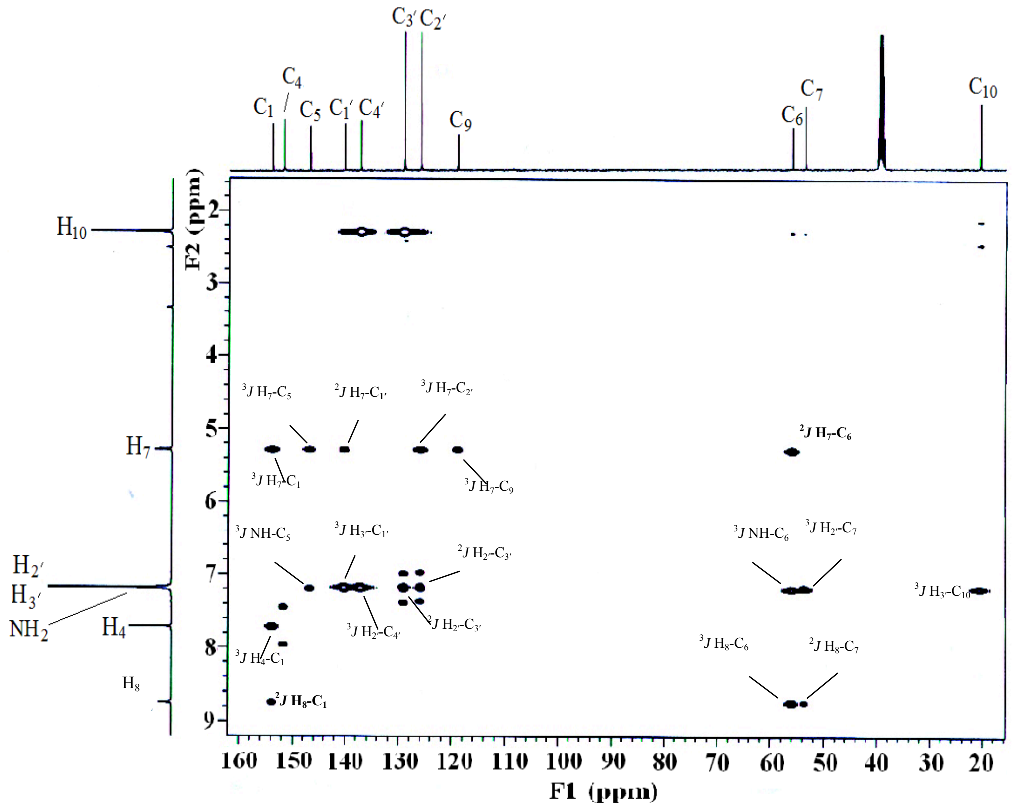

(**A**)

Figure S3. *Cont.*

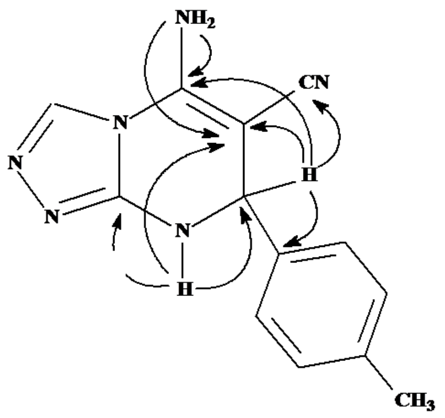

(**B**)

Figure S4. (**a**) The ^{13}C NMR spectrum of compound **4f** in DMSO; (**b**) The DEPT ^{13}C NMR spectrum of compound **4f** in DMSO.

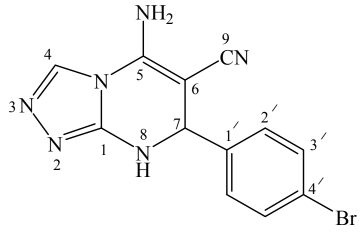

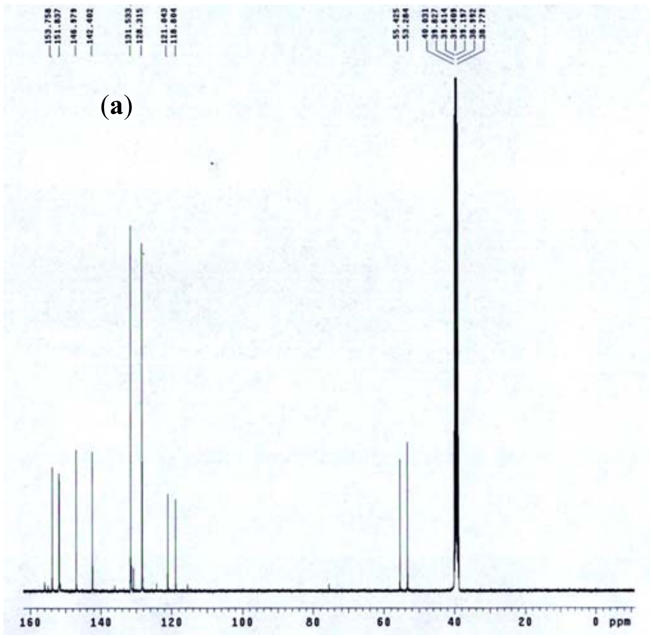

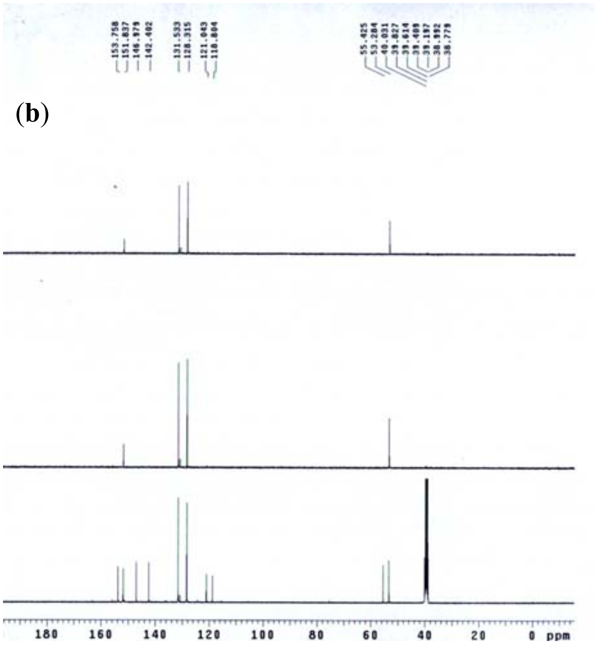

Figure S5. ^{1}H NM spectra of products; (**A**) ^{1}H-NMR spectrum of **4a**; (**B**) ^{1}H-NMR spectrum of **4b**; (**C**) ^{1}H-NMR spectrum of **4f**; (**D**) ^{1}H-NMR spectrum of **4g**; (**E**) ^{1}H-NMR spectrum of **4h**; (**F**) Partial enlarged spectrum of **4h**; (**G**) ^{1}H-NMR spectrum of **4i**; (**H**) ^{1}H-NMR spectrum of **4j**; (**I**) Partial enlarged spectrum of **4j**; (**J**) ^{1}H-NMR spectrum of **4j**; (**K**) ^{1}H-NMR spectrum of **4k**; (**L**) Partial enlarged spectrum of **4k**; (**M**) ^{1}H-NMR spectrum of **4l**; (**N**) ^{1}H-NMR spectrum of **4m**.

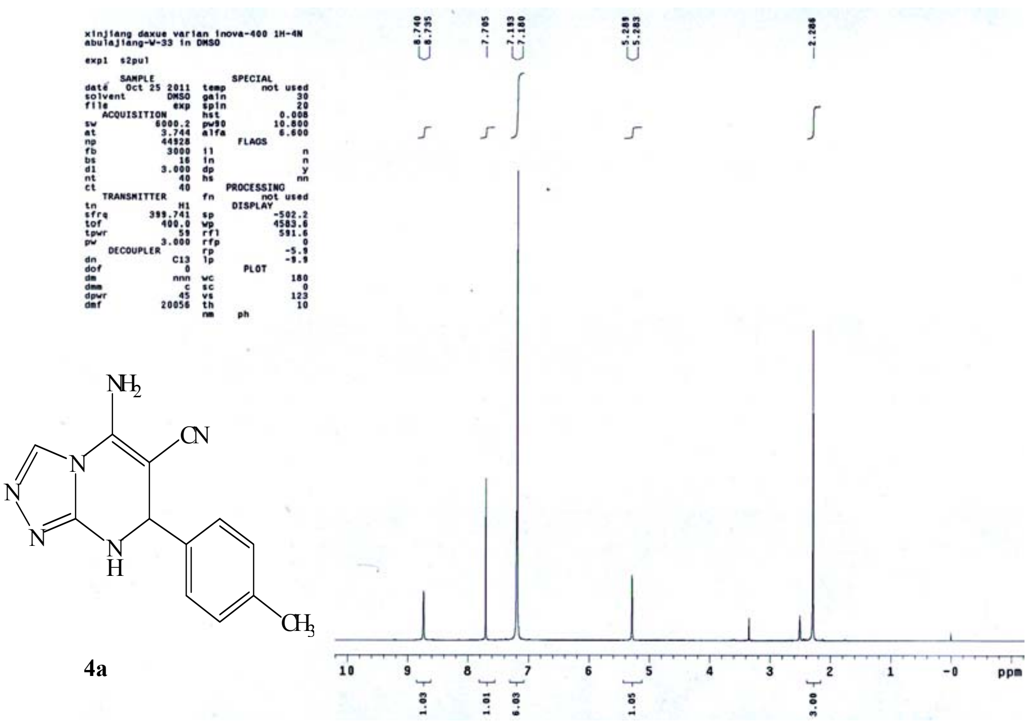

(**A**)

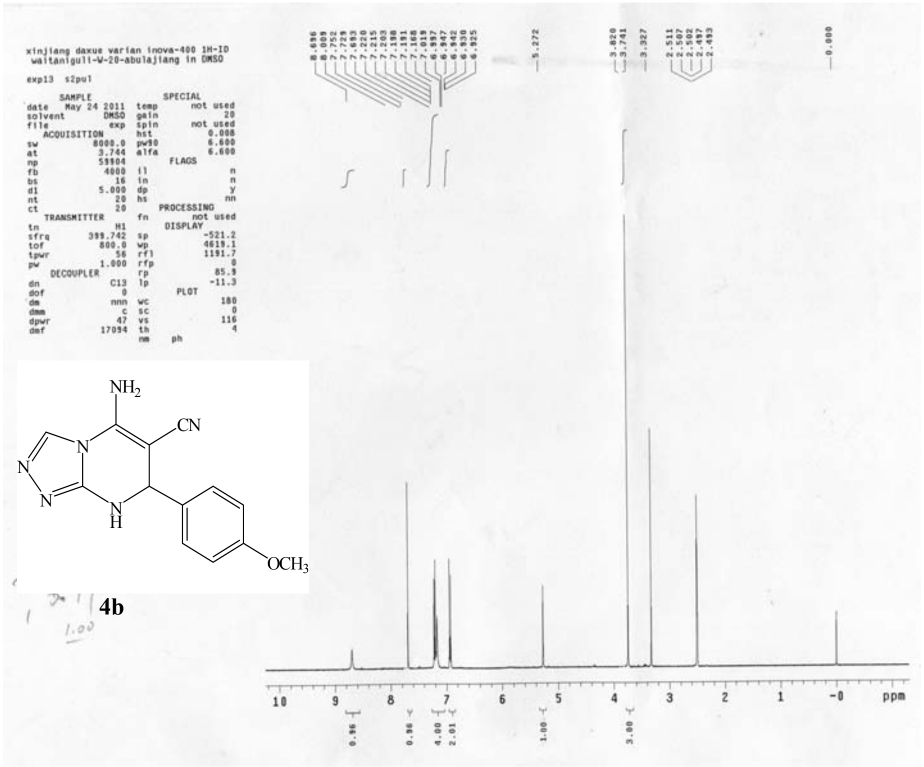

(**B**)

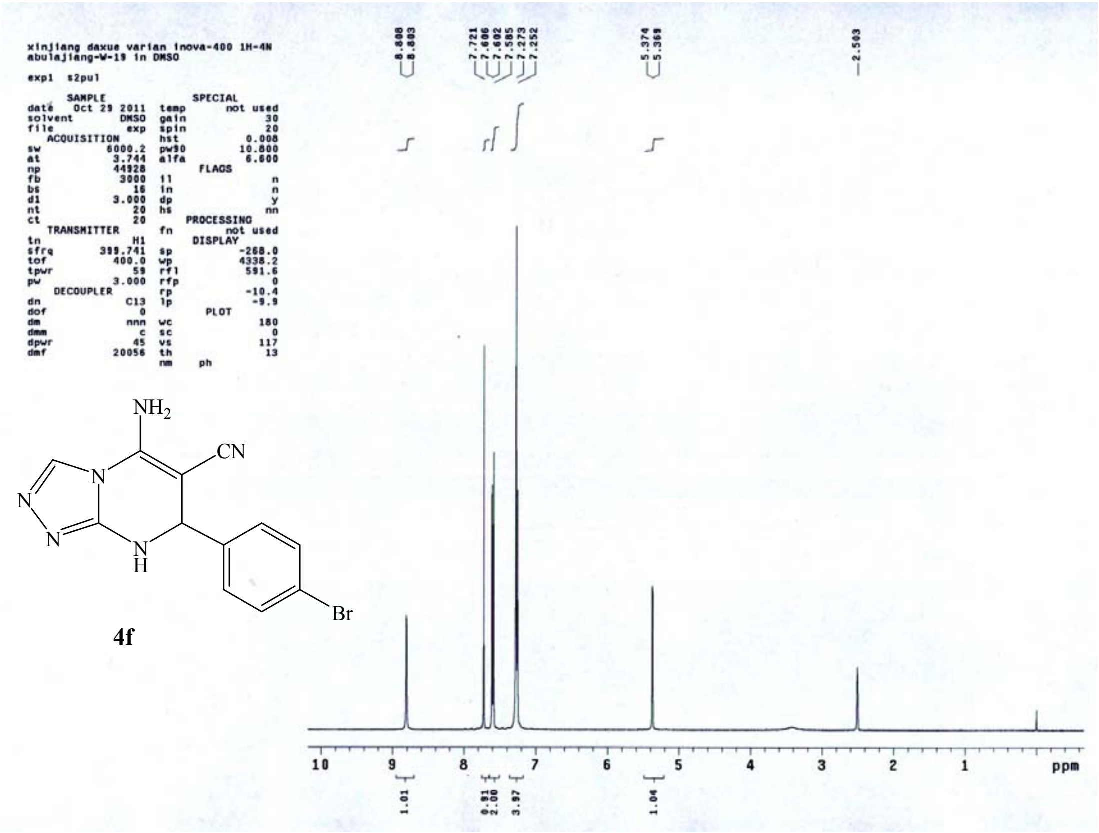

(**C**)

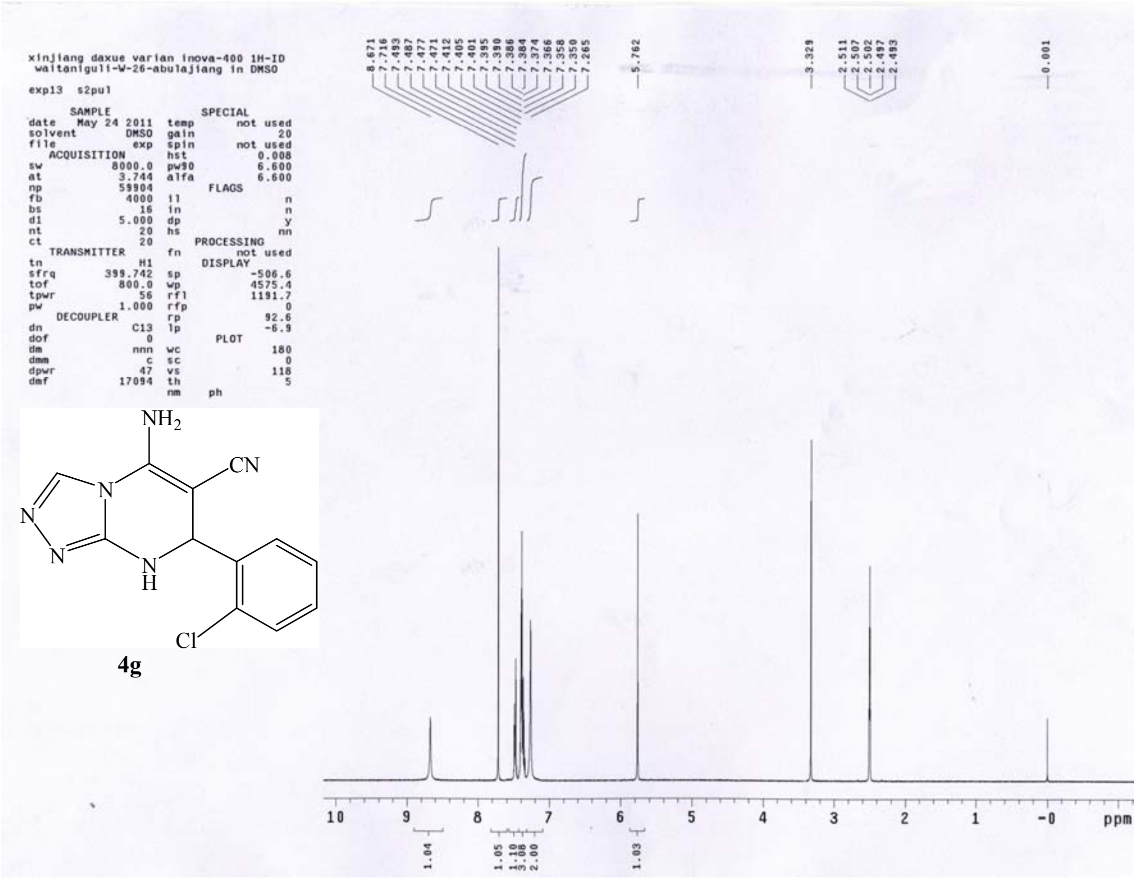

(**D**)

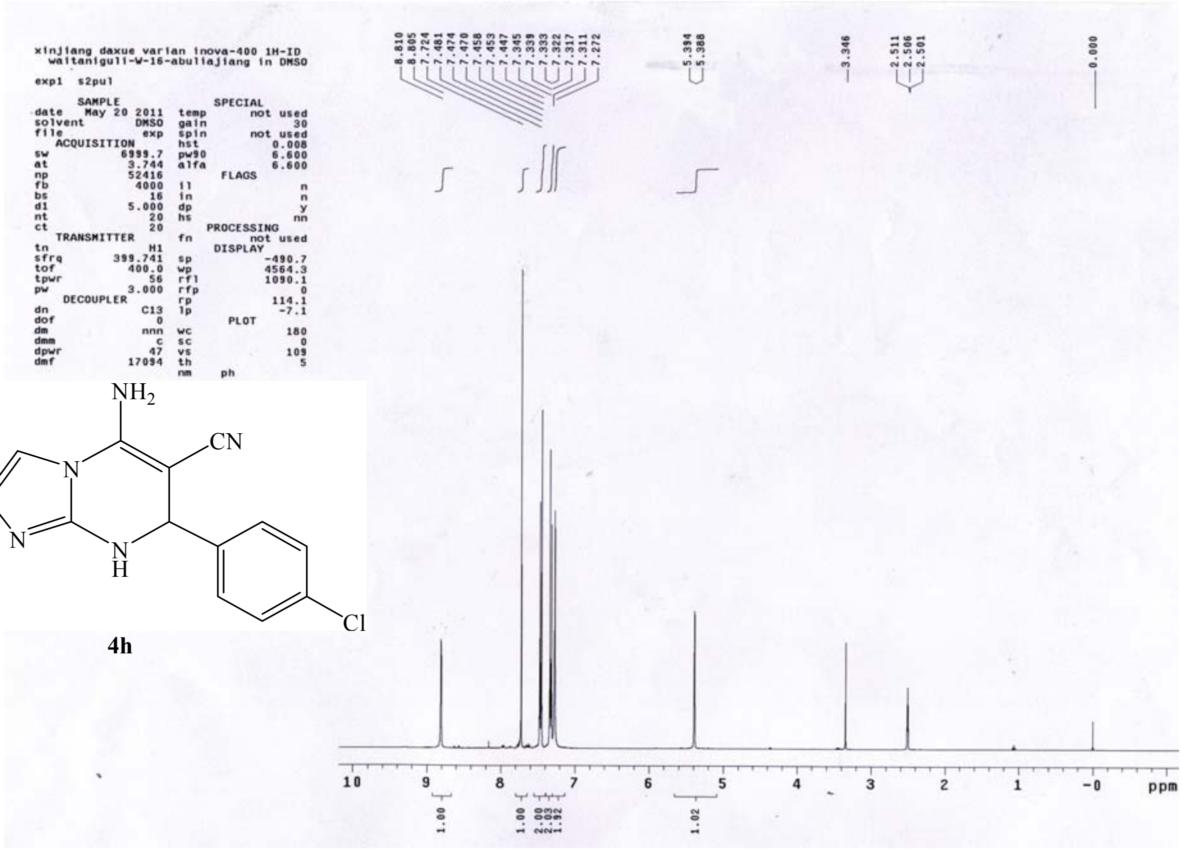

(**E**)

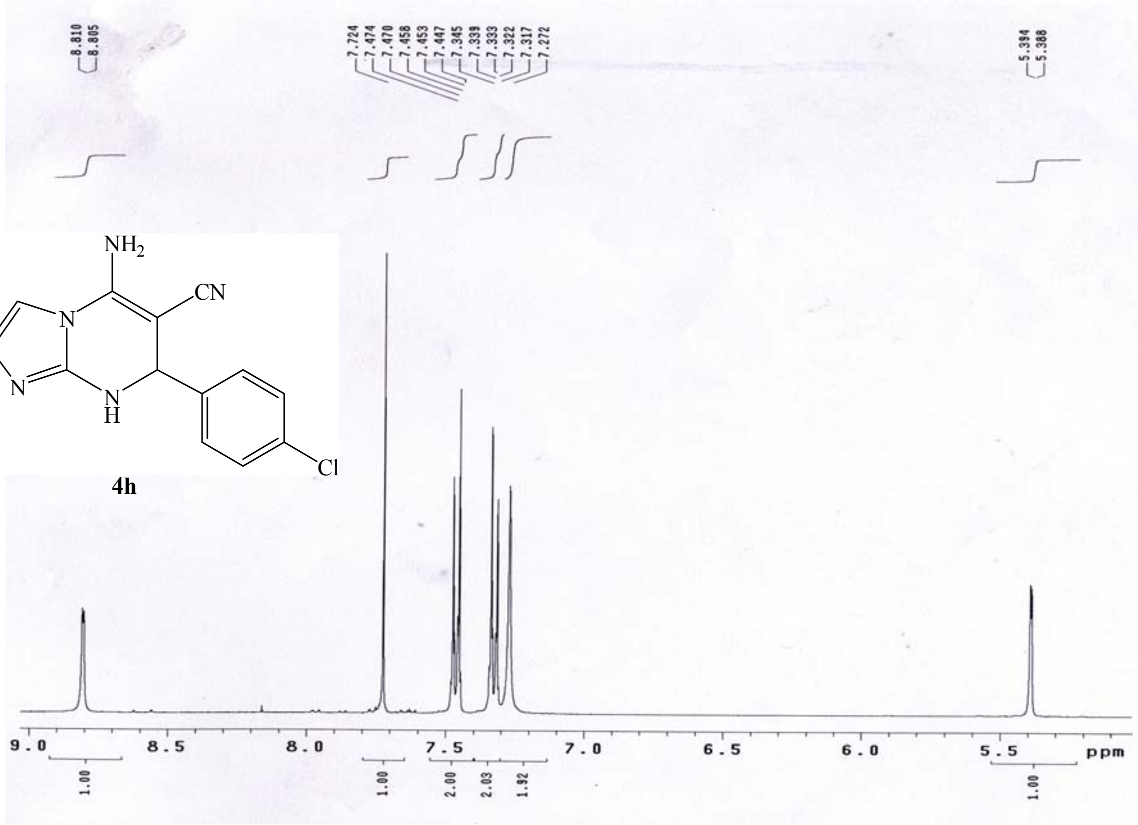

(**F**)

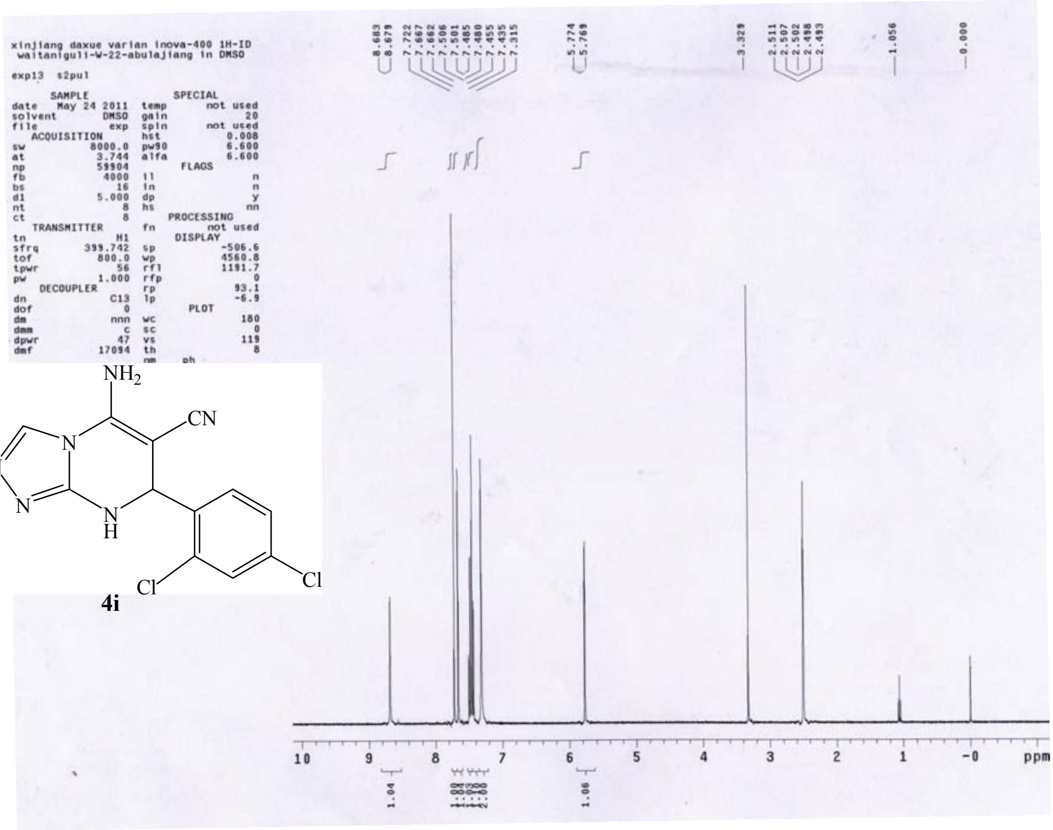
xinjiang daxue varian inova-400 1H-ID
waitaniguli-W-22-abulajiang in DMSO
exp13 s2pul
NH2
CN
N
N
N
N
H
Cl
Cl
4i
ppm

(**G**)

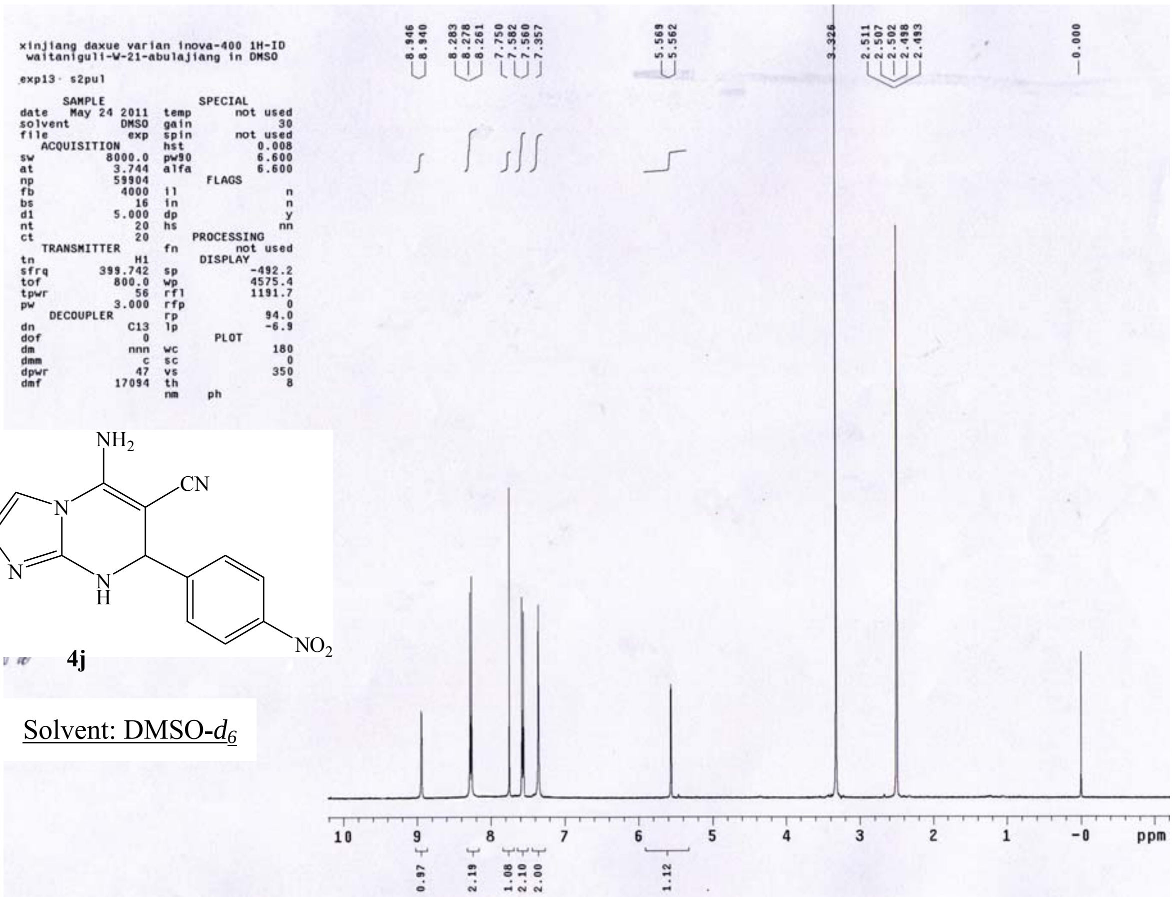
xinjiang daxue varian inova-400 1H-ID
waitaniguli-W-21-abulajiang in DMSO
exp13 s2pul
NH2
CN
N
N
N
N
H
NO2
4j
Solvent: DMSO-d_6
ppm

(**H**)

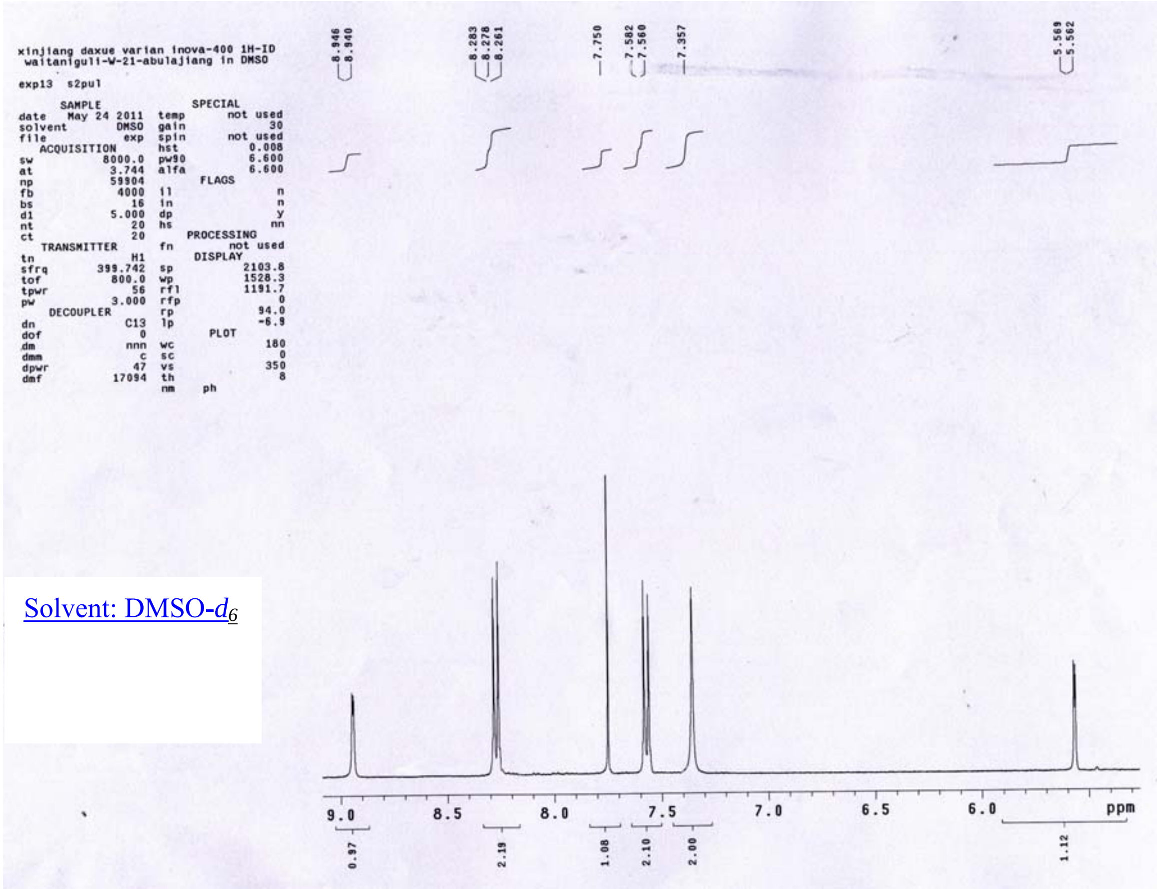

(I)

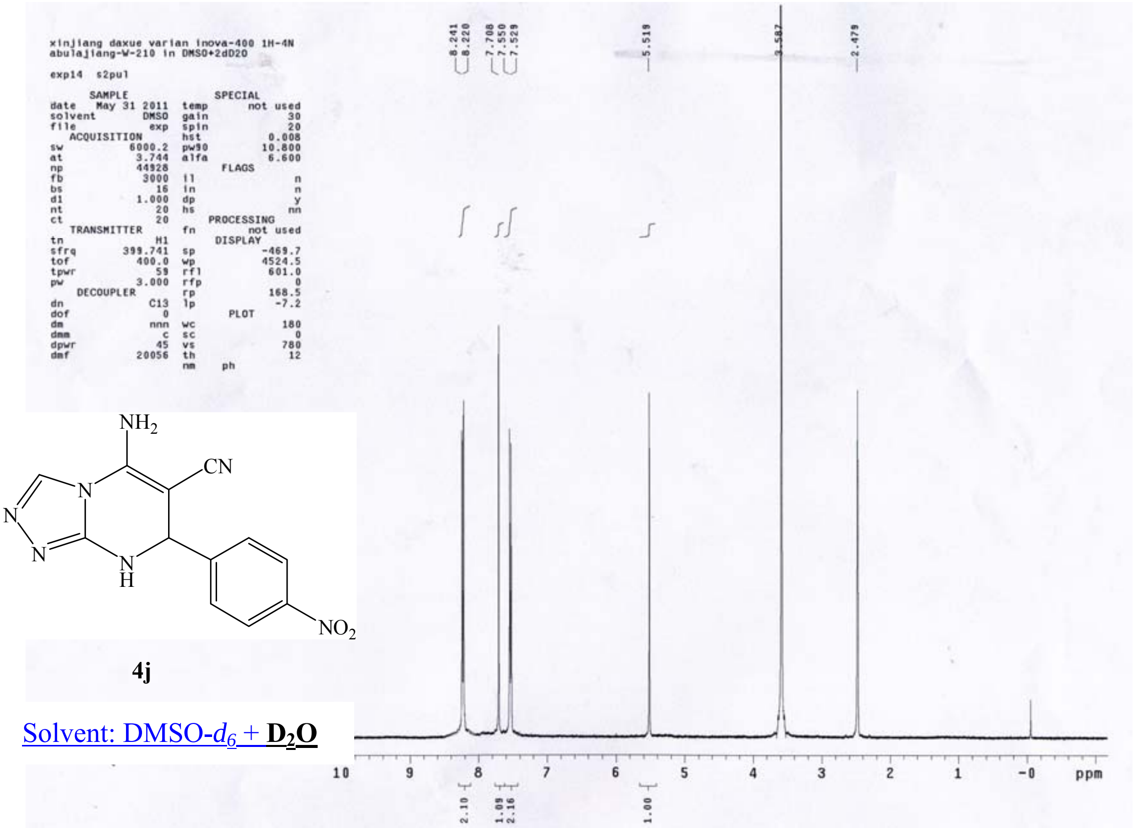

(J)

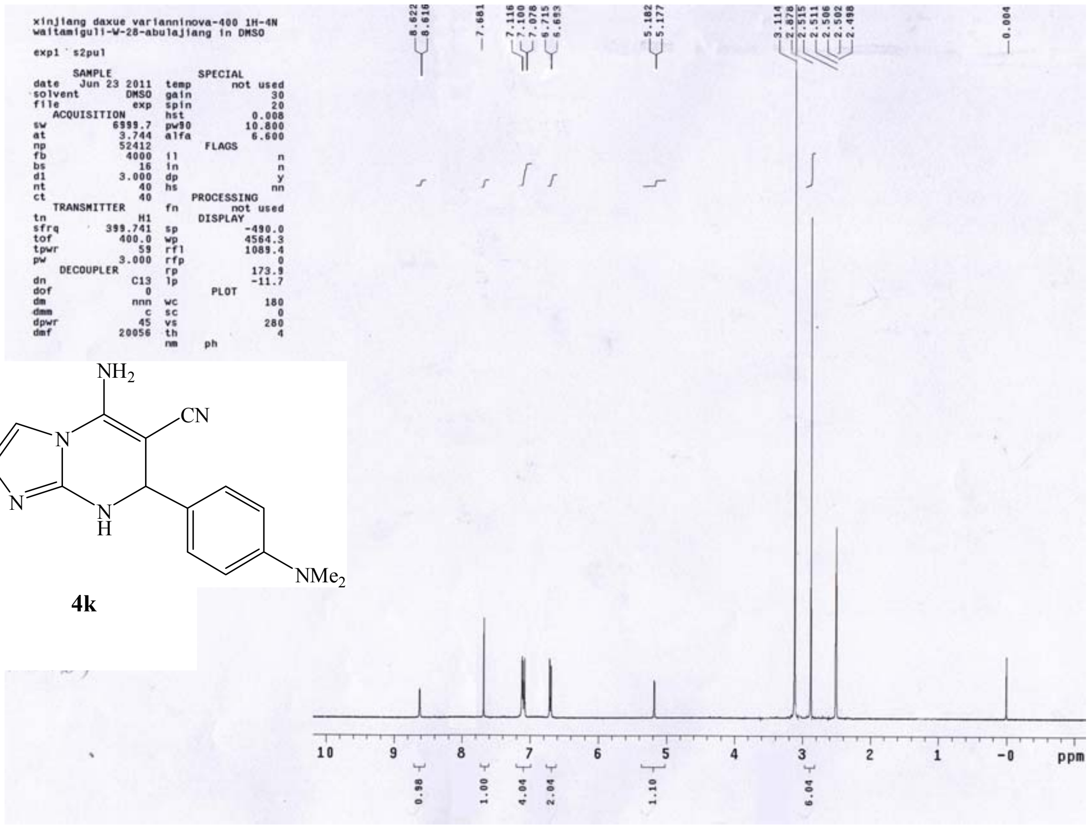
xinjiang daxue varianninova-400 1H-4N
waitamiguli-W-28-abulajiang in DMSO
exp1 s2pul
SAMPLE
date Jun 23 2011
solvent DMSO
file exp
ACQUISITION
sw 6999.7
at 3.744
np 52412
fb 4000
bs 16
d1 3.000
nt 40
ct 40
TRANSMITTER
tn H1
sfrq 399.741
tof 400.0
tpwr 59
pw 3.000
DECOUPLER
dn C13
dof 0
dm nnn
dmm c
dpwr 45
dmf 20056
SPECIAL
temp not used
gain 30
spin 20
hst 0.008
pw90 10.800
alfa 6.600
FLAGS
il n
in n
dp y
hs nn
PROCESSING
fn not used
DISPLAY
sp -490.0
wp 4564.3
rfl 1089.4
rfp 0
rp 173.9
lp -11.7
PLOT
wc 180
sc 0
vs 280
th 4
nm ph
8.622
8.616
7.681
7.116
7.100
7.078
6.715
6.693
5.182
5.177
3.114
2.878
2.515
2.511
2.506
2.502
2.498
0.004
NH_2
CN
N
N
N
N
H
NMe_2
4k
10 9 8 7 6 5 4 3 2 1 -0 ppm
0.98
1.00
4.04
2.04
1.10
6.04

(**K**)

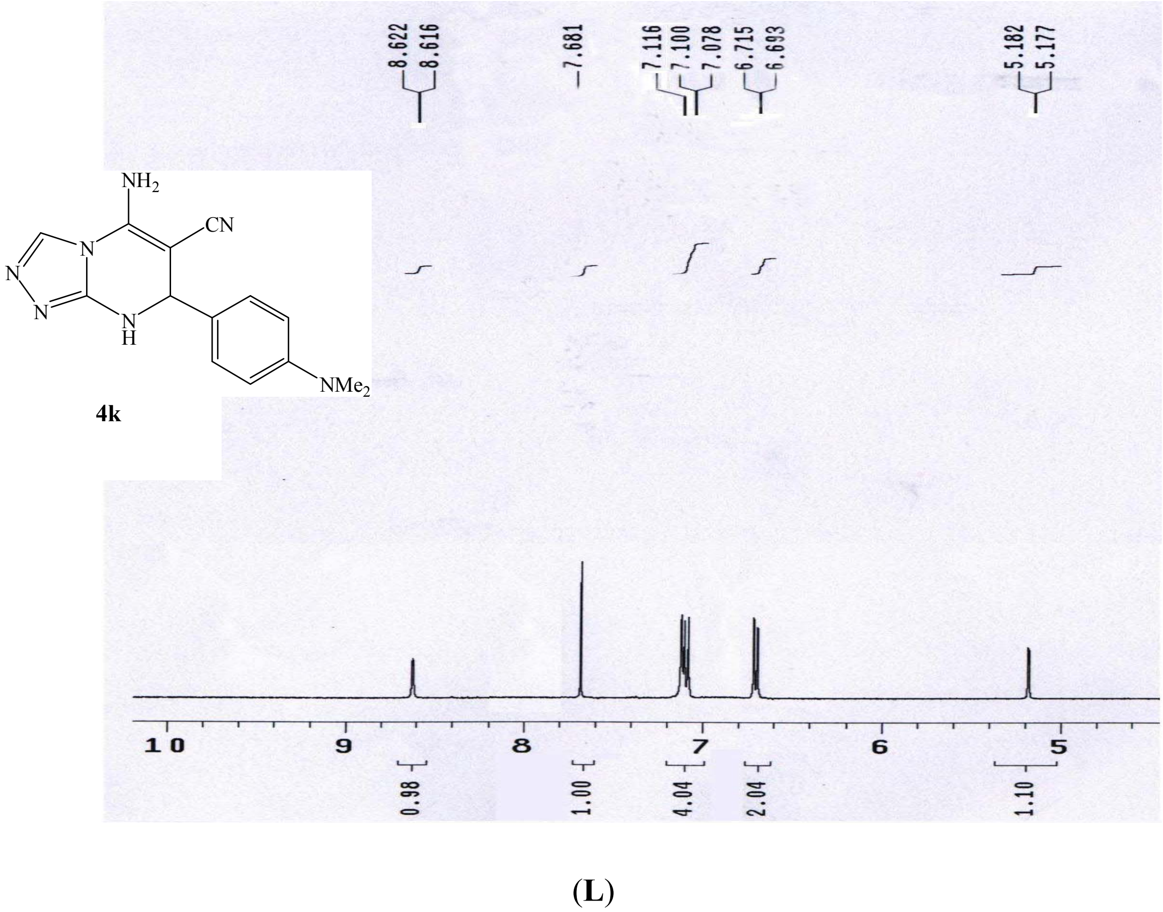
8.622
8.616
7.681
7.116
7.100
7.078
6.715
6.693
5.182
5.177
NH_2
CN
N
N
N
N
H
NMe_2
4k
10 9 8 7 6 5
0.98
1.00
4.04
2.04
1.10

(**L**)

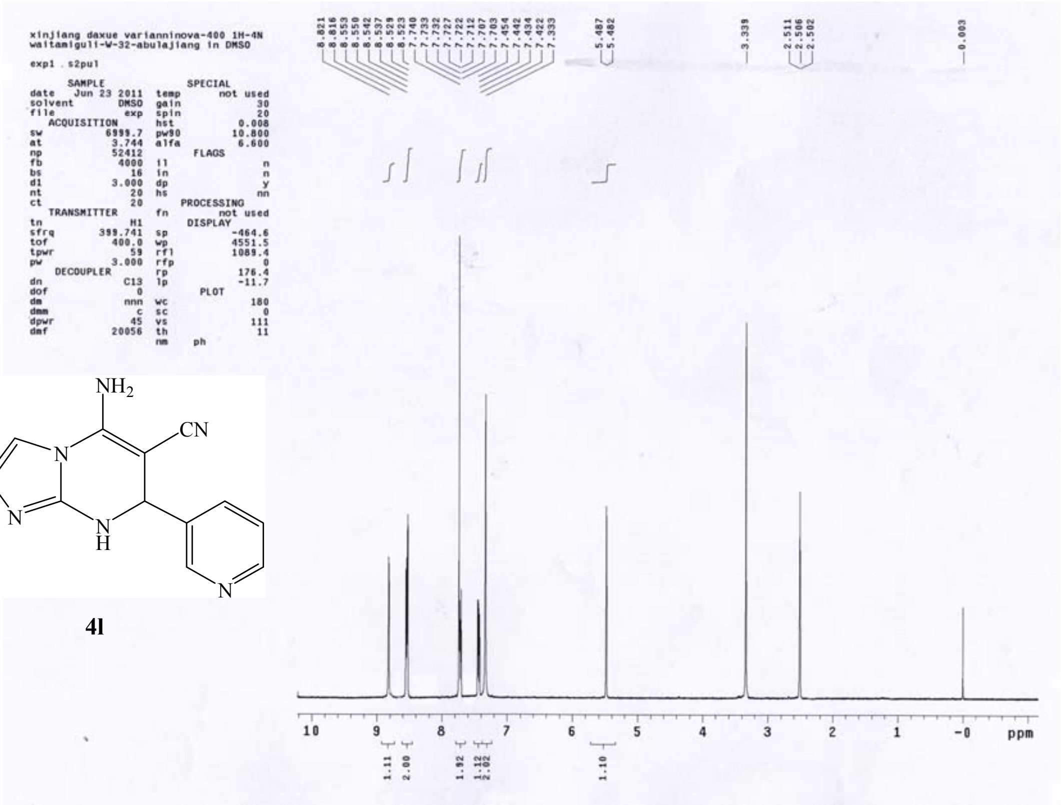

4l

(**M**)

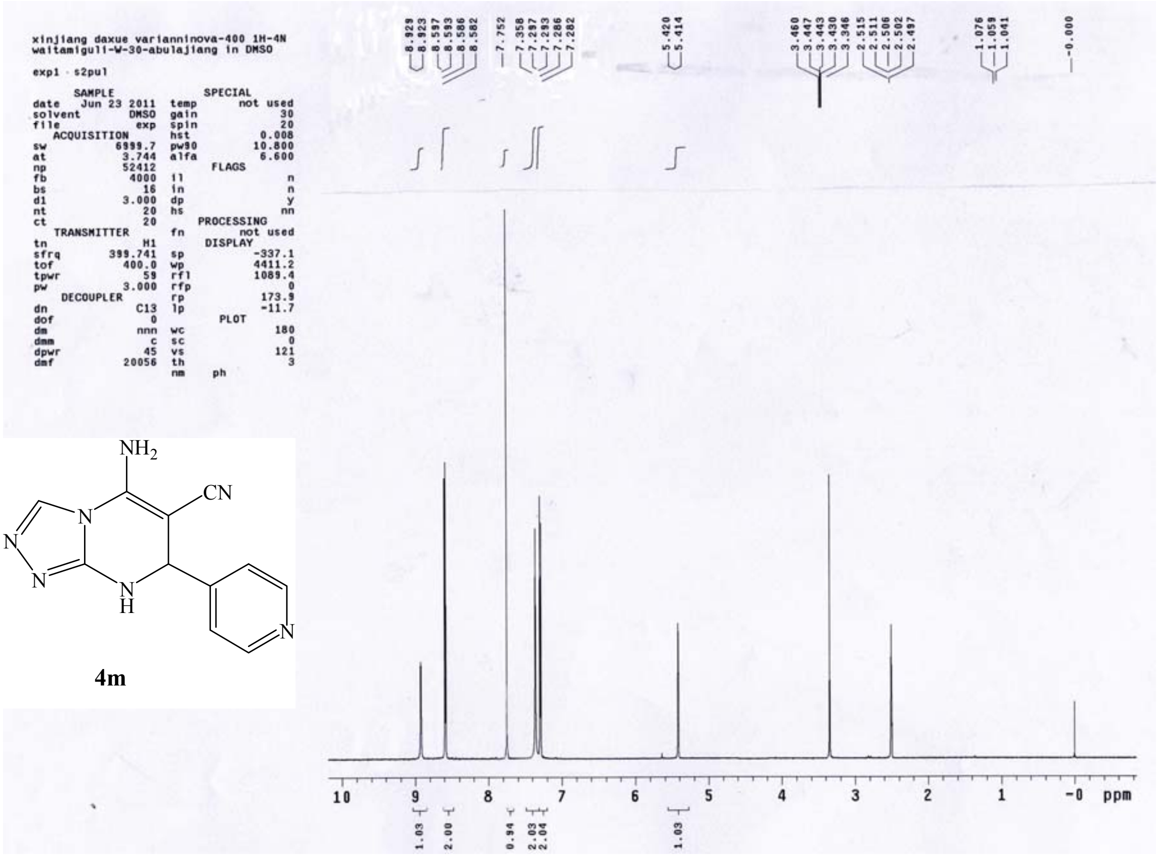

4m

(**N**)

Figure 6. 13C NMR spectra of products.

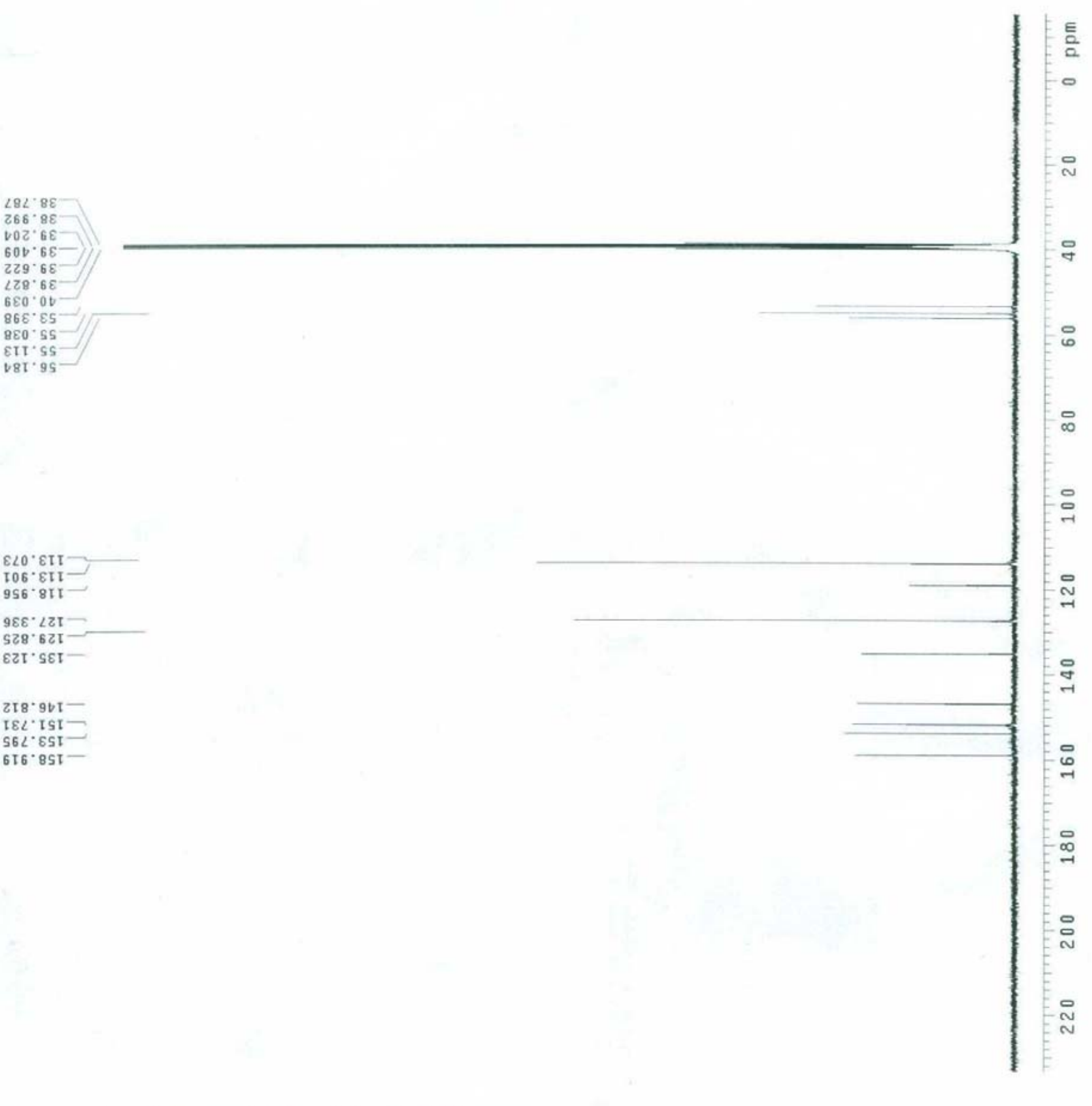

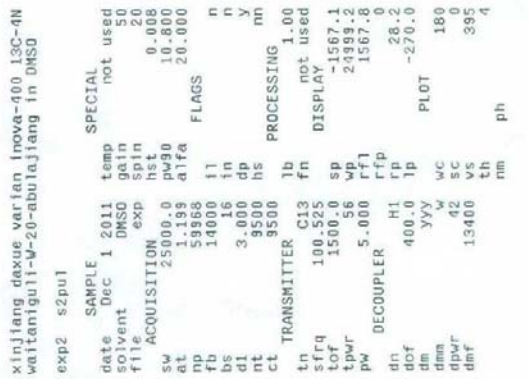

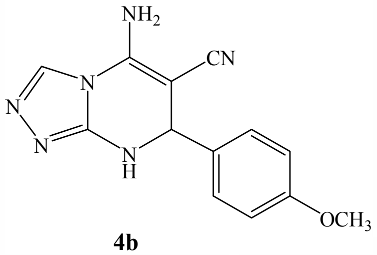

4b

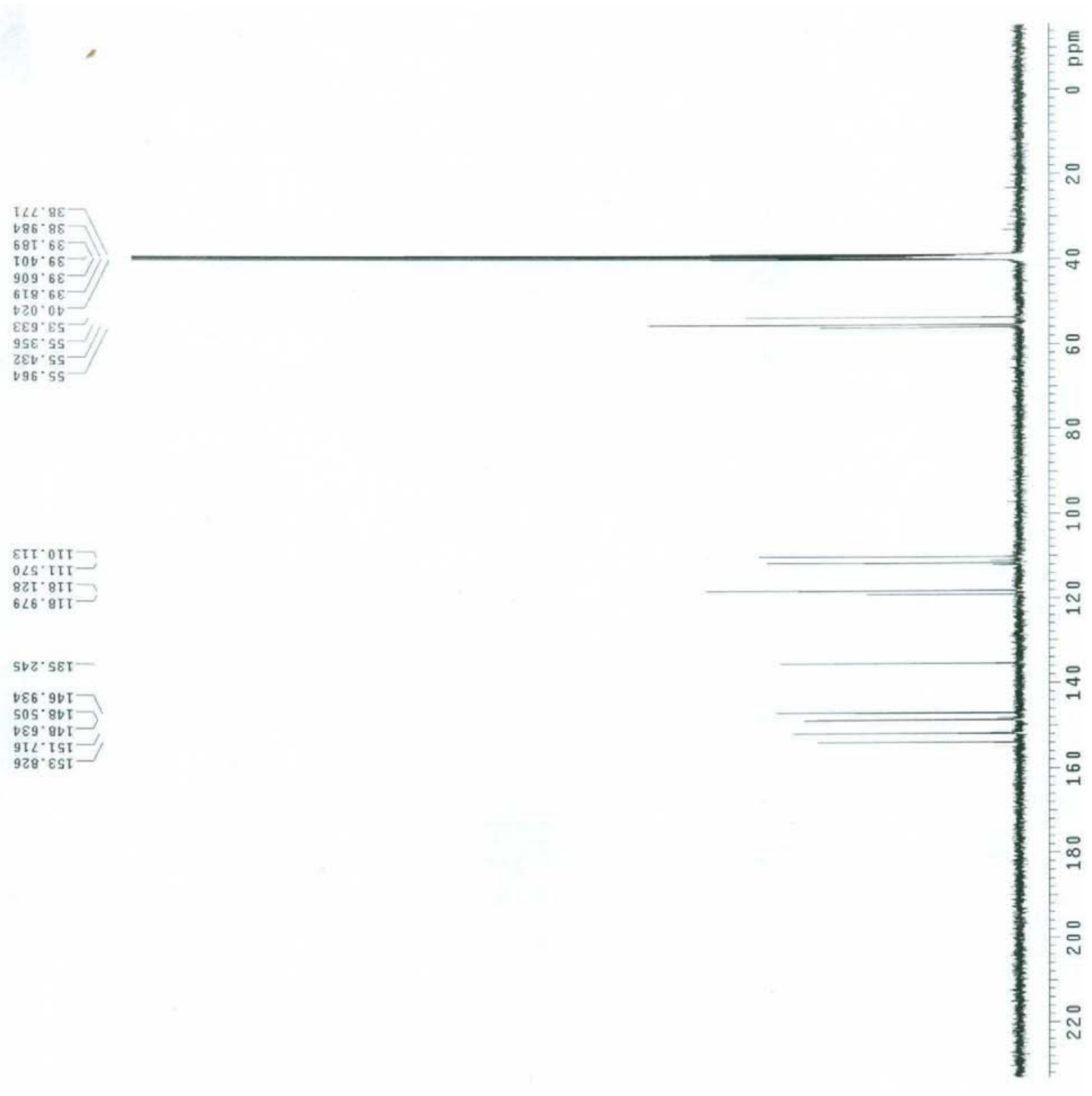
38.771
38.984
39.189
39.401
39.606
39.819
40.024
53.633
55.356
55.432
55.964
110.113
111.570
118.128
118.979
135.245
146.934
148.505
148.634
151.716
153.826
ppm

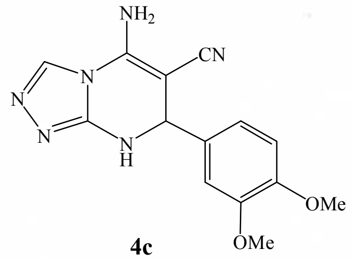
NH2
CN
N
N
N
N
H
OMe
OMe

4c

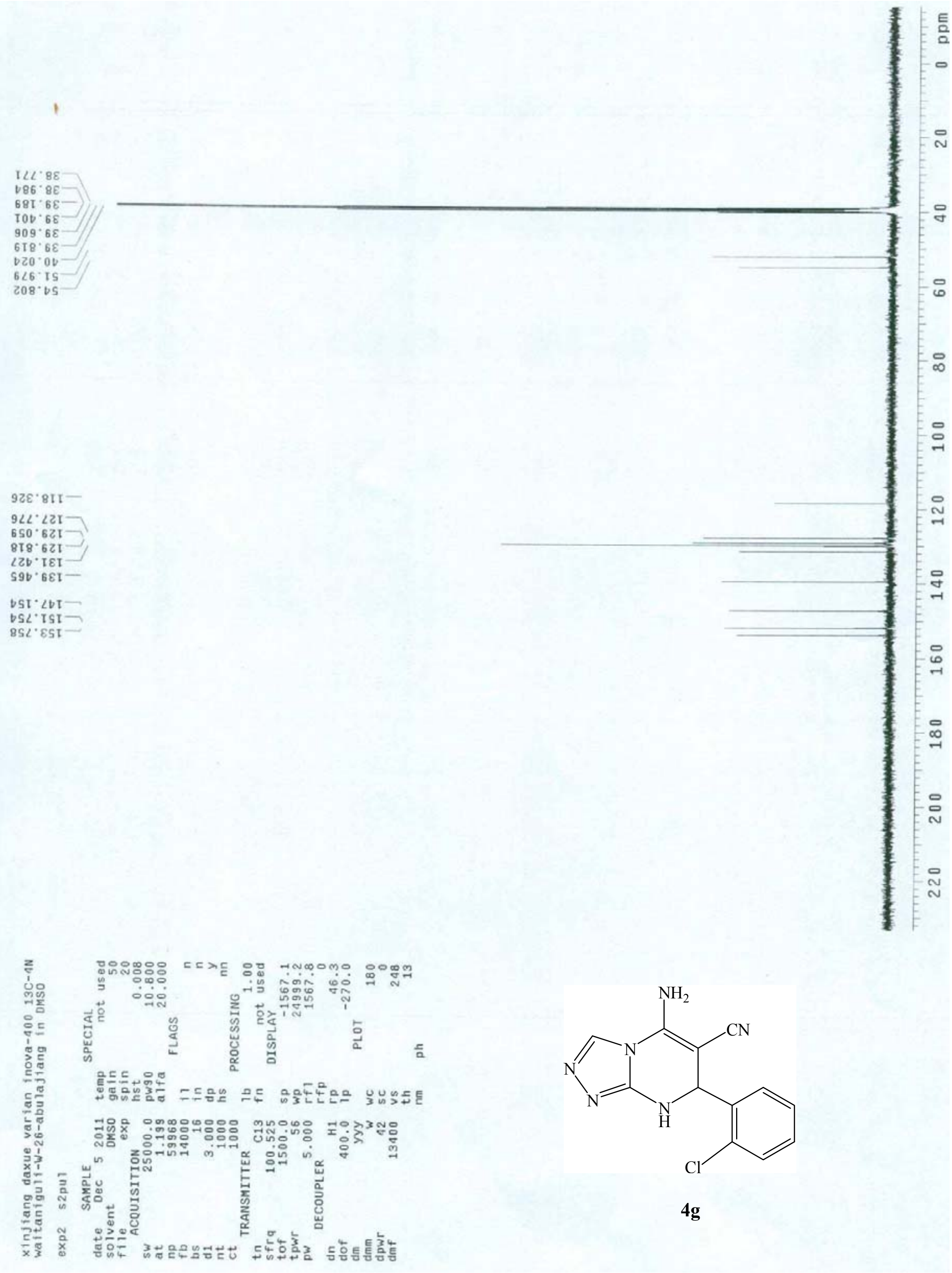
38.771
38.984
39.189
39.401
39.606
39.819
40.024
51.979
54.802
118.326
127.776
129.059
129.818
131.427
139.465
147.154
151.754
153.758
ppm
NH2
CN
N
N
N
H
Cl

4g

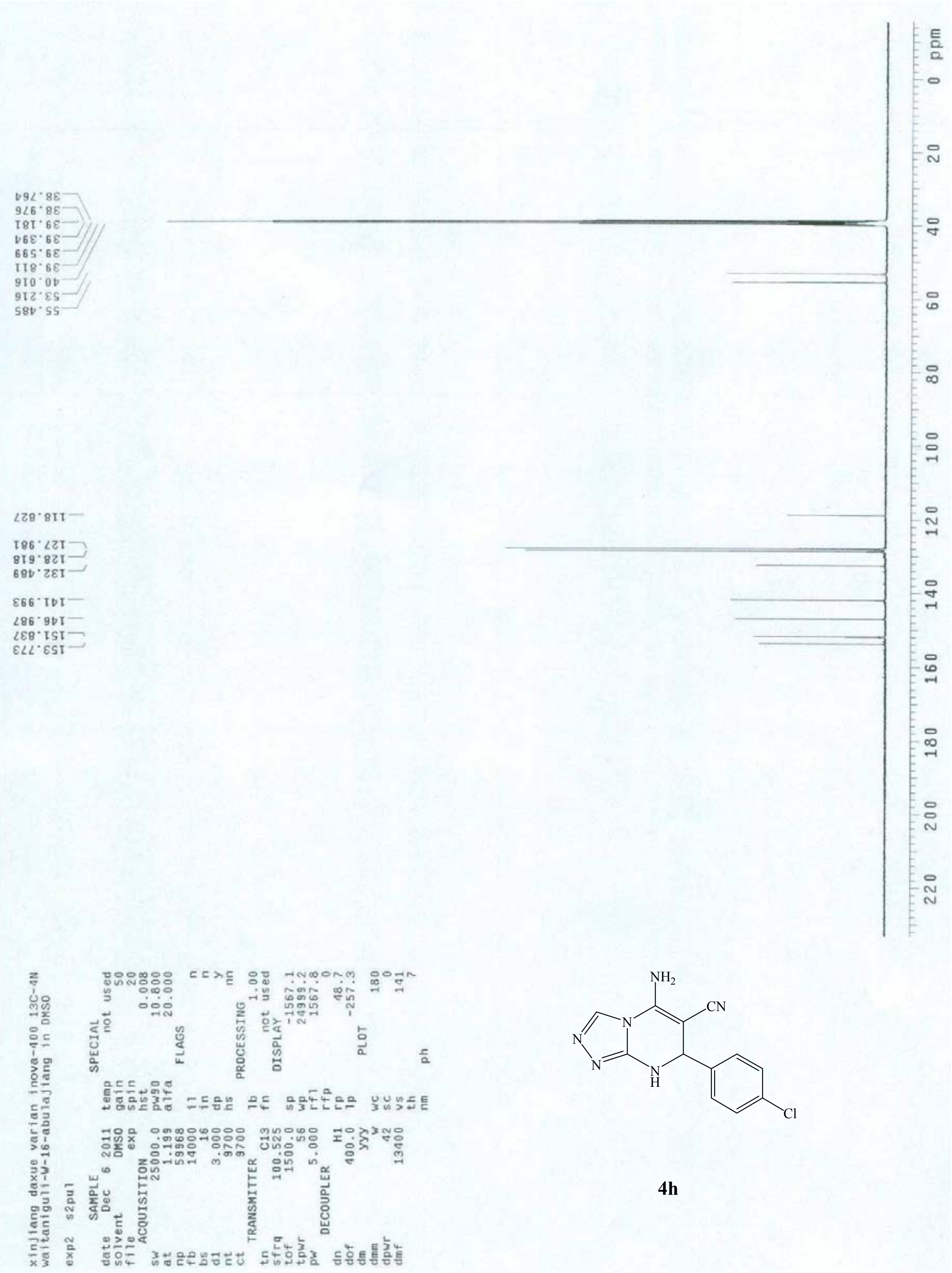
xinjiang daxue varian inova-400 13C-4N
waitaniguli-W-16-abulajiang in DMSO
38.764
38.976
39.181
39.394
39.599
39.811
40.016
53.216
55.485
118.827
127.981
128.618
132.489
141.993
146.987
151.837
153.773
ppm
NH_2
CN
N
N
N
H
Cl

4h

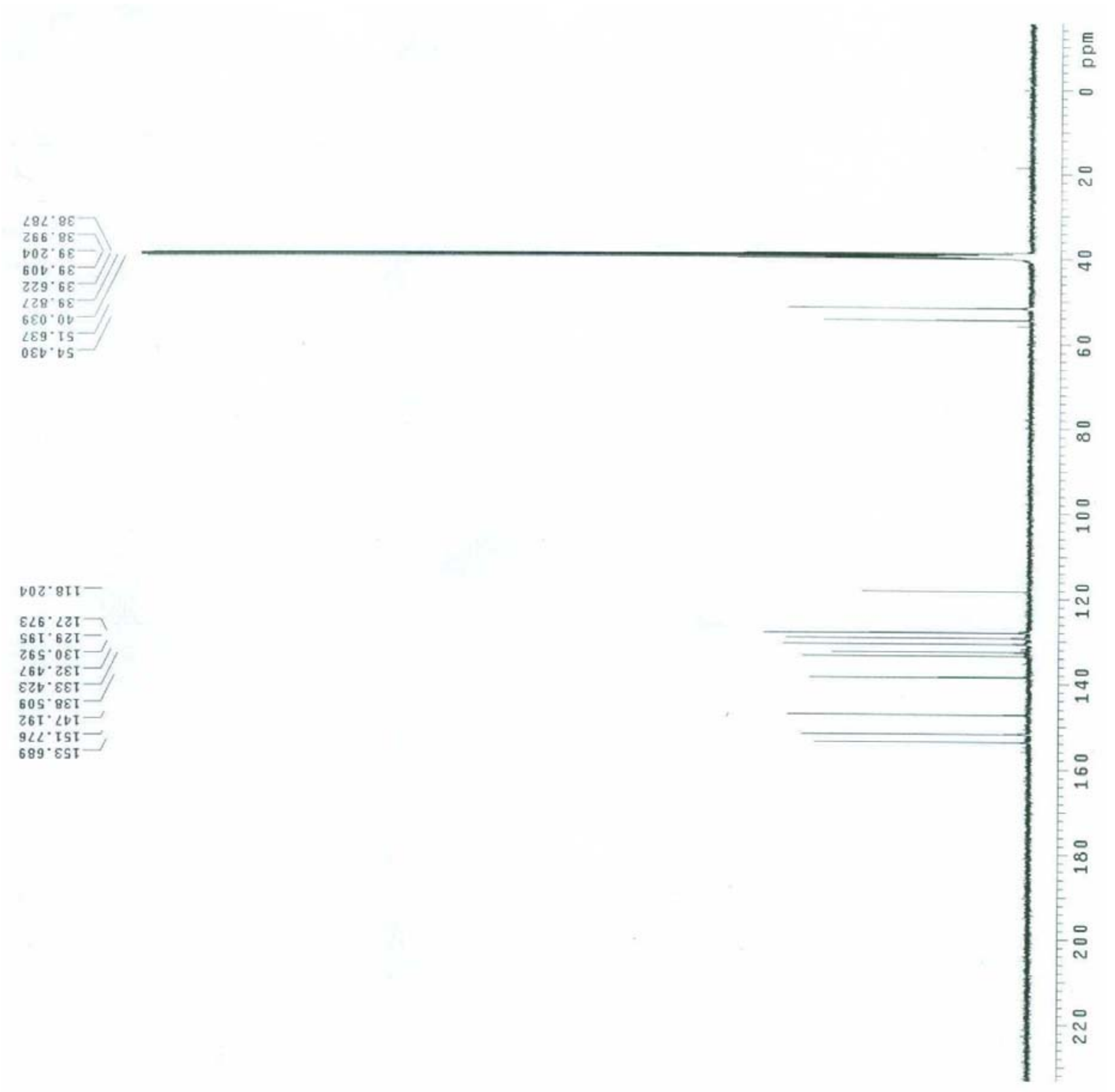

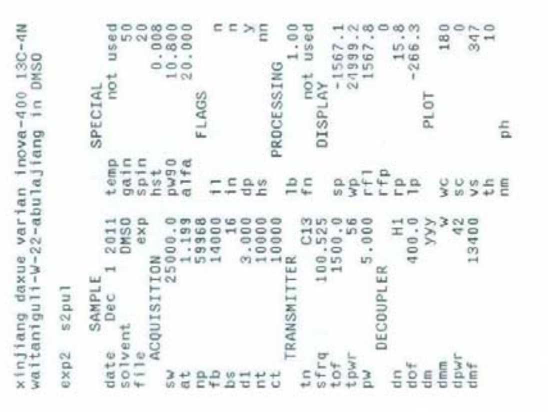

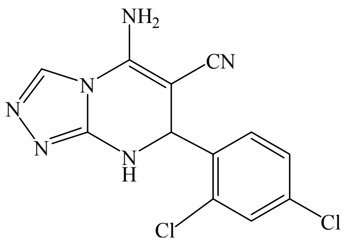

4i

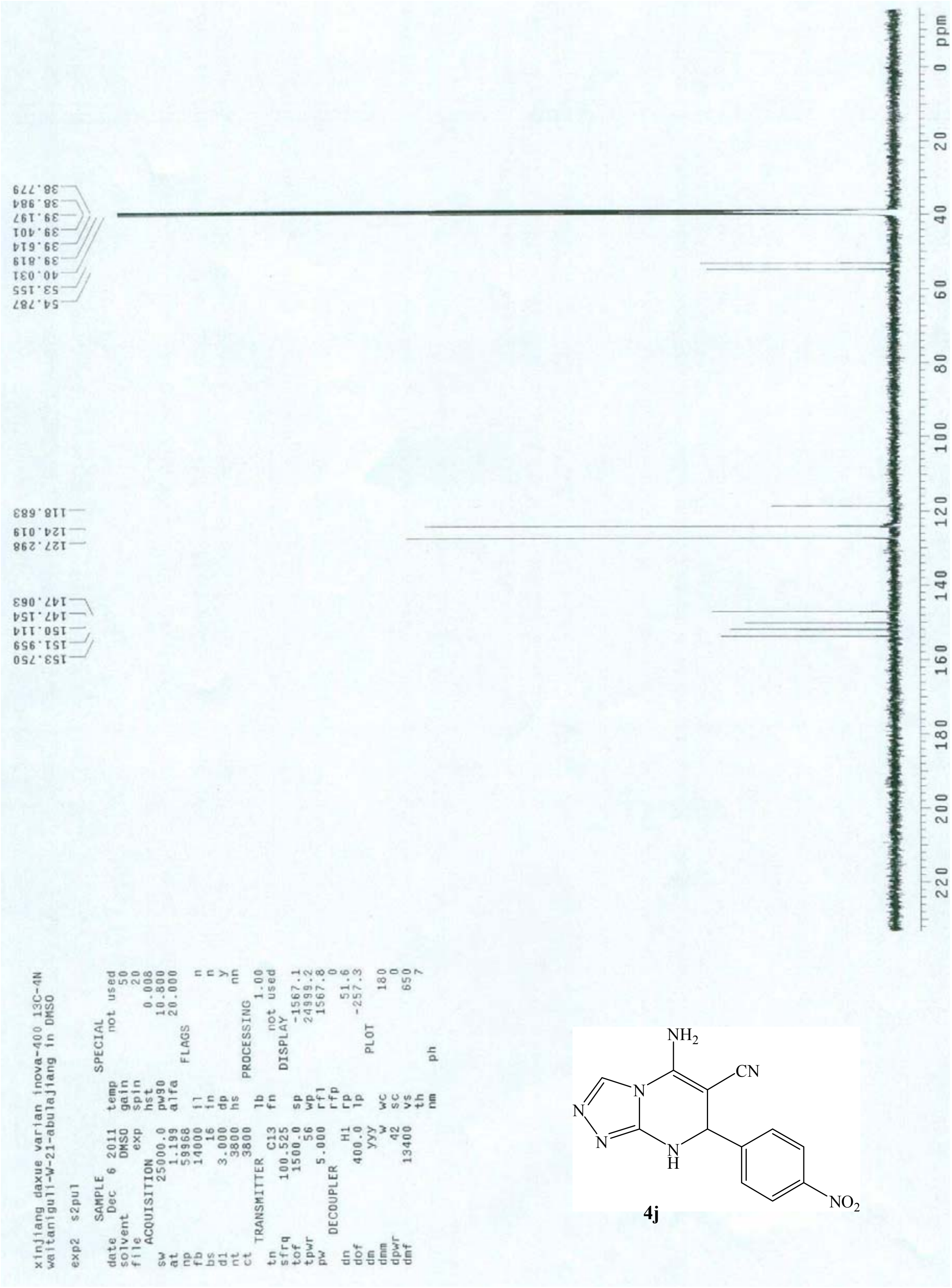
38.779
38.984
39.197
39.401
39.614
39.819
40.031
53.155
54.787
118.683
124.019
127.298
147.063
147.154
150.114
151.959
153.750
ppm
0 20 40 60 80 100 120 140 160 180 200 220
NH_2
CN
N
H
NO_2
4j

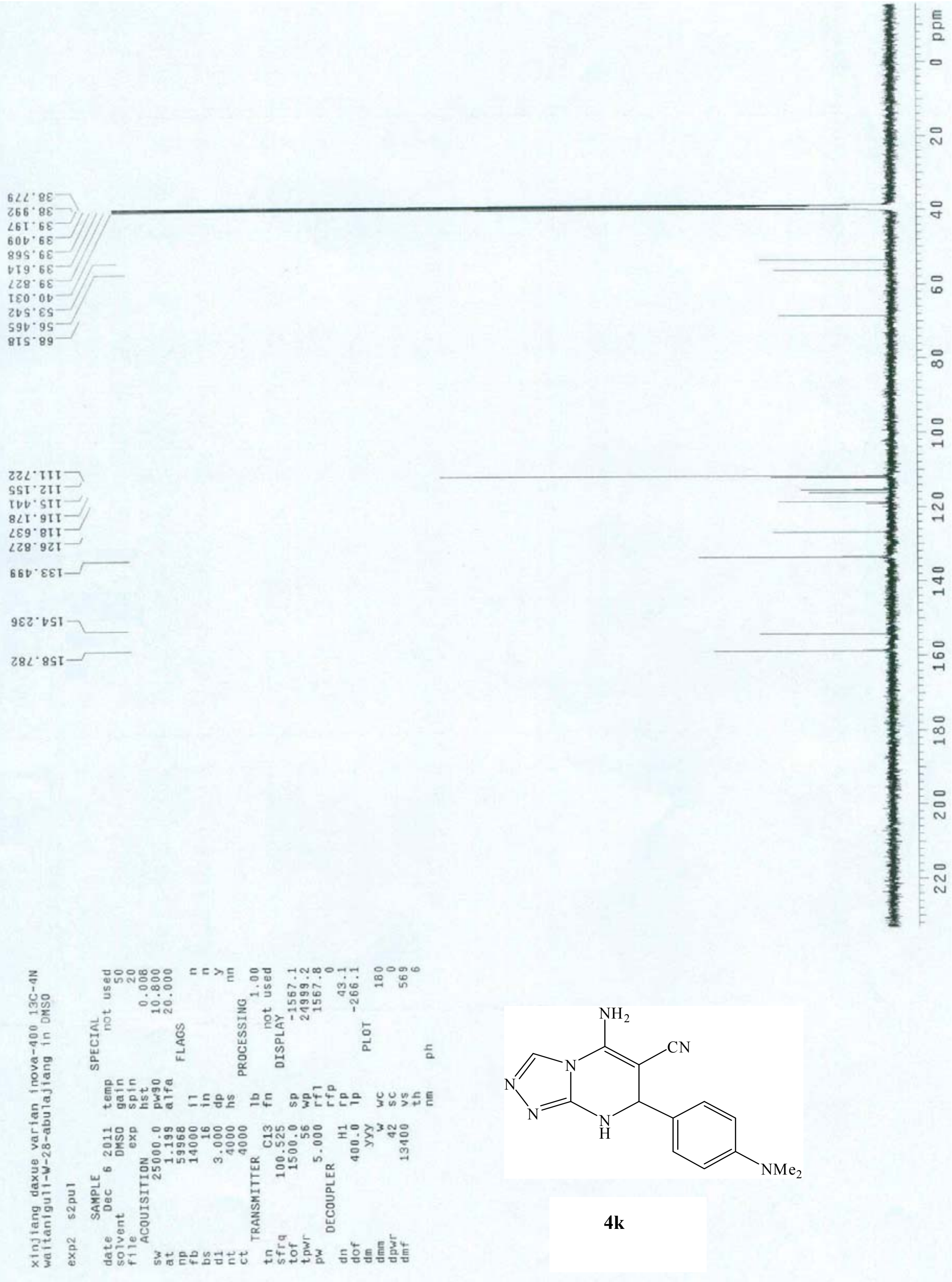
158.782
154.236
133.499
126.827
118.637
116.178
115.441
112.155
111.722
68.518
56.465
53.542
40.031
39.827
39.614
39.568
39.409
39.197
38.992
38.779
ppm
NH2
CN
NMe2
4k

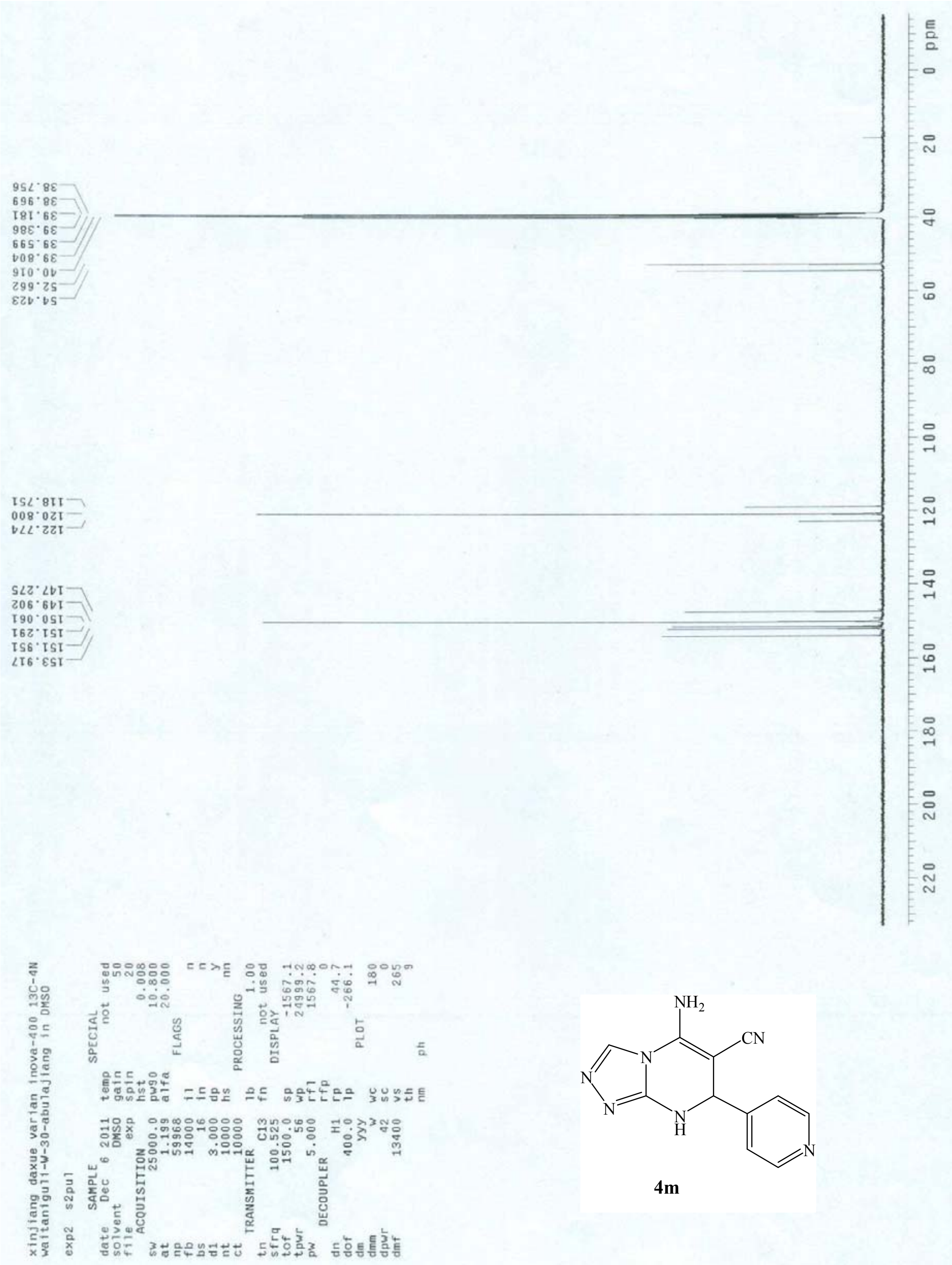
38.756
38.969
39.181
39.386
39.599
39.804
40.016
52.662
54.423
118.751
120.800
122.774
147.275
149.902
150.061
151.291
151.951
153.917
ppm
NH_2
CN
N
H
4m

Figure S7. IR spectra of products. (**A**) IR spectrum of **4a**; (**B**) IR spectrum of **4b**; (**C**) IR spectrum of **4c**; (**D**) IR spectrum of **4e**; (**E**) IR spectrum of **4f**; (**F**) IR spectrum of **4g**; (**G**) IR spectrum of **4h**; (**H**) IR spectrum of **4i**; (**I**) IR spectrum of **4j**; (**J**) IR spectrum of **4l**.

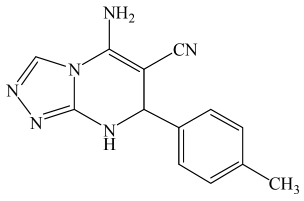

4a

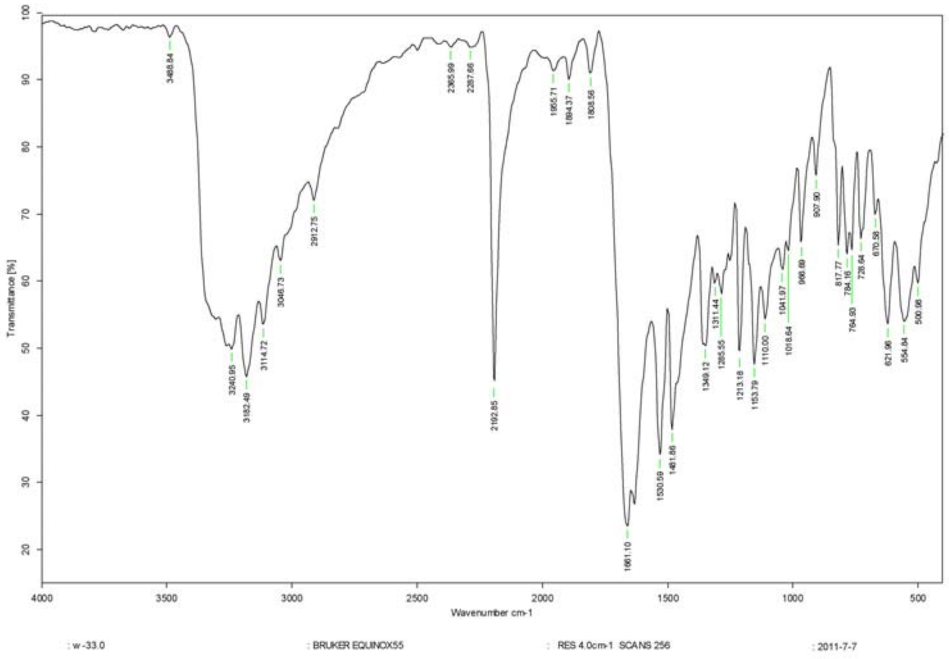

(**A**)

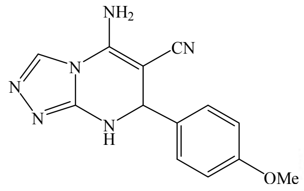

4b

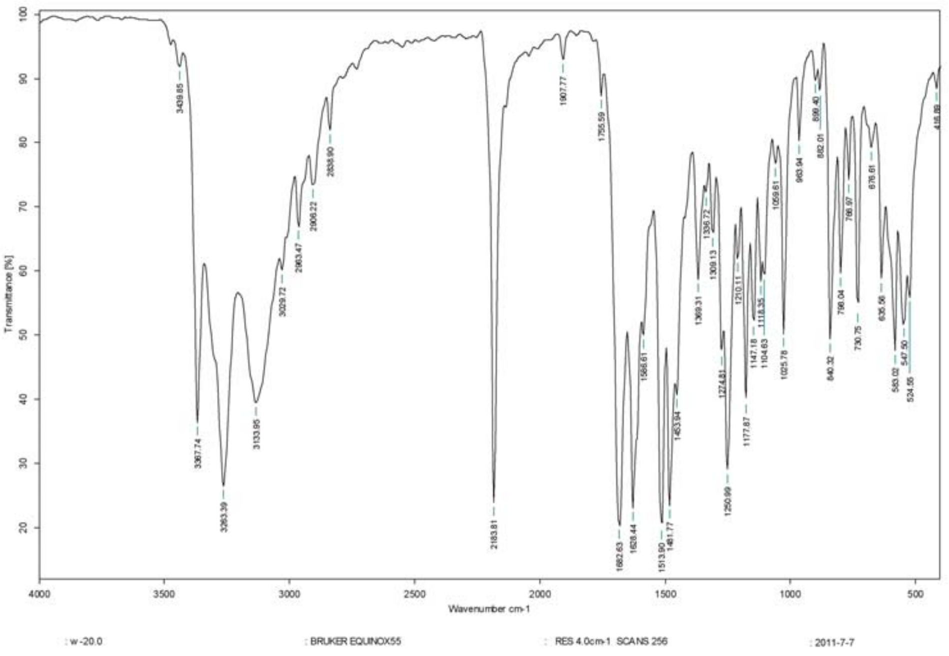

(**B**)

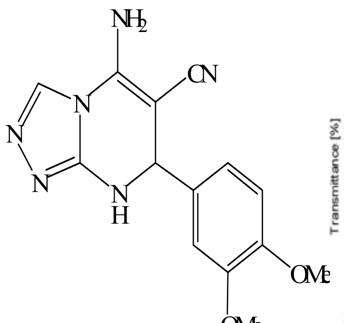
NH2
CN
N
N
N
N
H
OMe
OMe

4c

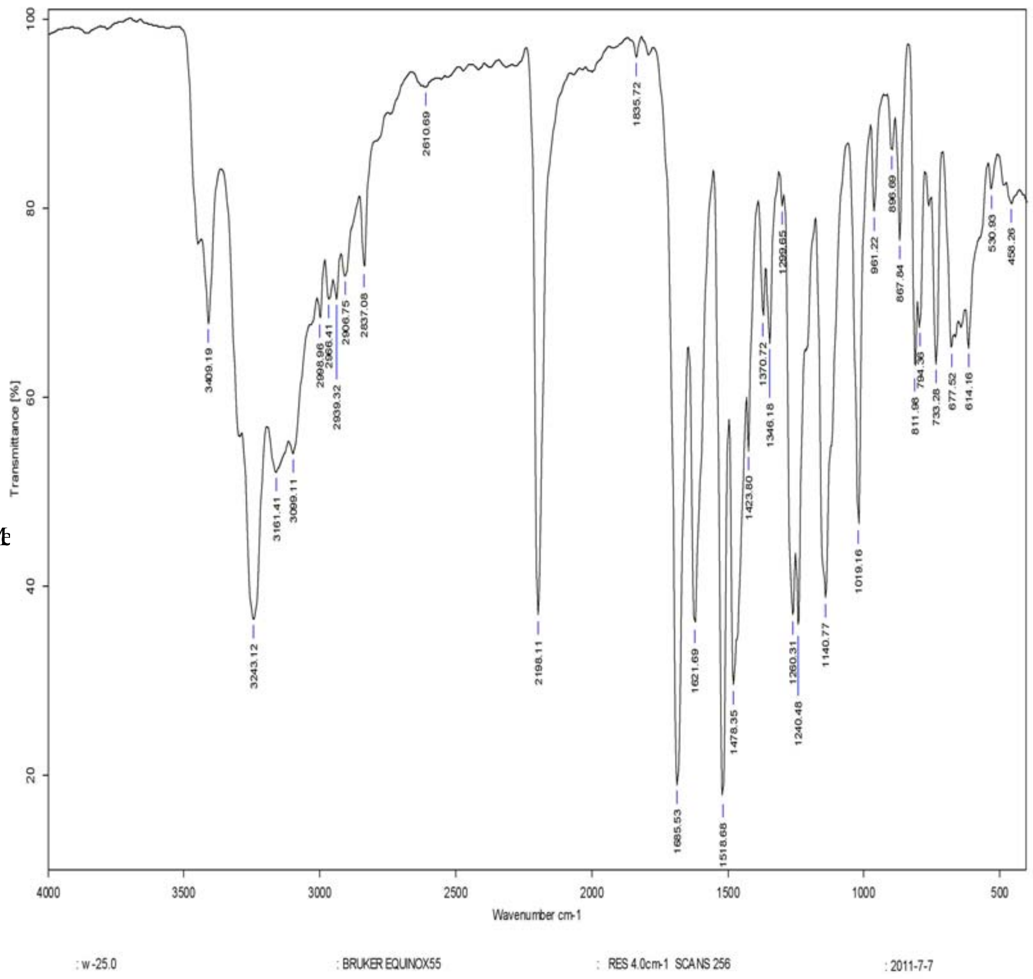
Transmittance [%]
Wavenumber cm-1
: w -25.0
: BRUKER EQUINOX55
: RES 4.0cm-1 SCANS 256
: 2011-7-7

(**C**)

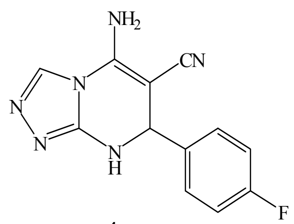
NH2
CN
N
N
N
N
H
F

4e

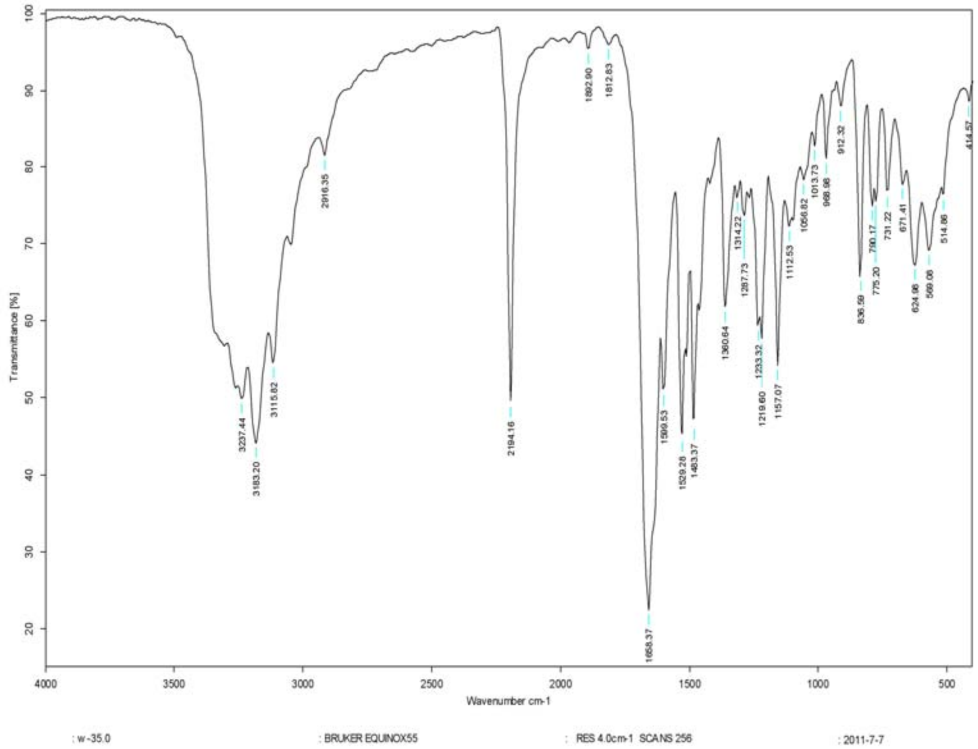
Transmittance [%]
Wavenumber cm-1
: w -35.0
: BRUKER EQUINOX55
: RES 4.0cm-1 SCANS 256
: 2011-7-7

(**D**)

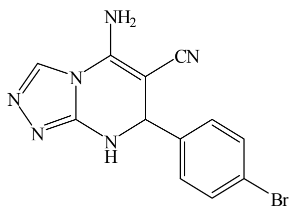
NH2
CN
N
N
N
N
H
Br

4f

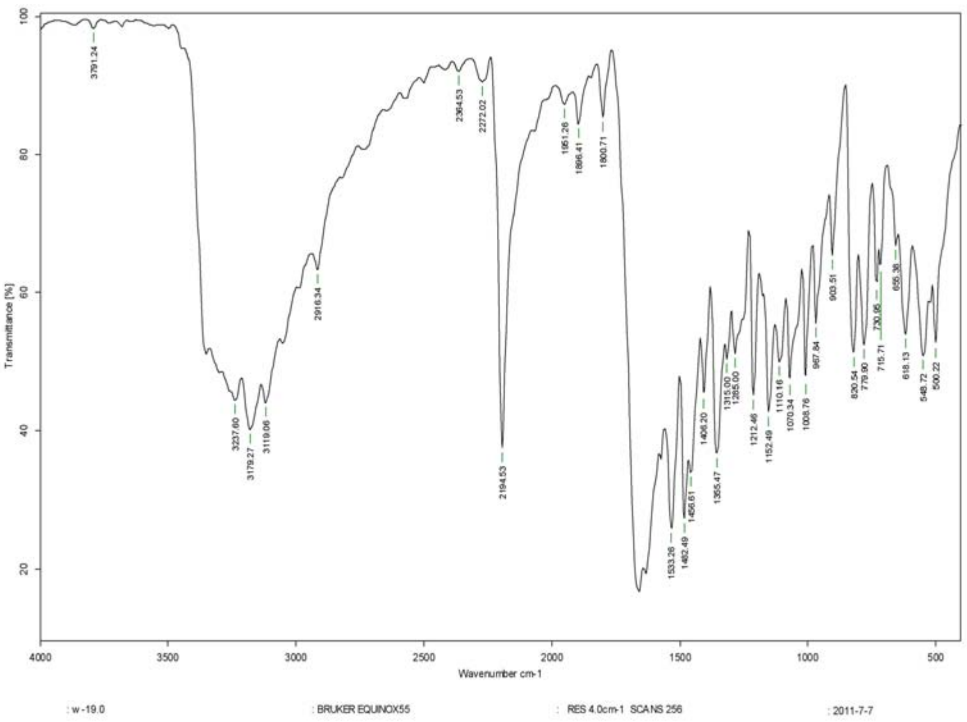
Transmittance [%]
Wavenumber cm-1
3791.24
3237.60
3179.27
3119.06
2916.34
2364.53
2272.02
2194.53
1951.26
1866.41
1800.71
1533.26
1482.49
1456.61
1406.20
1355.47
1315.00
1285.00
1212.46
1152.49
1110.16
1070.34
1008.76
967.84
903.51
830.54
779.90
730.95
715.71
655.38
618.13
548.72
500.22
: w-19.0
: BRUKER EQUINOX55
: RES 4.0cm-1 SCANS 256
: 2011-7-7

(**E**)

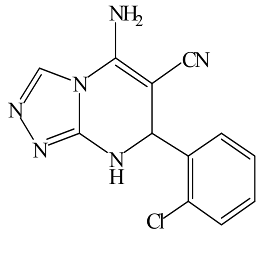
NH2
CN
N
N
N
N
H
Cl

4g

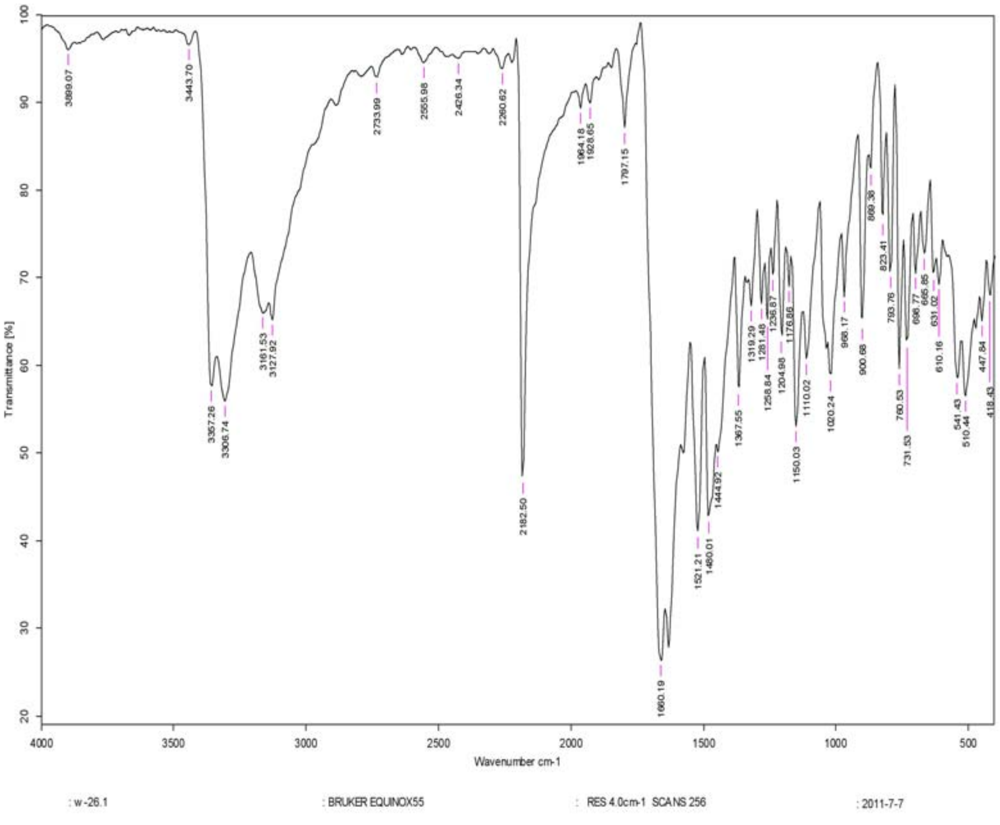
Transmittance [%]
Wavenumber cm-1
3899.07
3443.70
3357.26
3306.74
3161.53
3127.92
2733.99
2555.98
2426.34
2260.62
2182.50
1964.18
1928.65
1797.15
1660.19
1521.21
1480.01
1444.92
1367.55
1319.29
1281.48
1258.84
1236.87
1204.98
1176.86
1150.03
1110.02
1020.24
968.17
900.66
869.38
823.41
793.76
760.53
731.53
696.77
665.85
631.02
610.16
541.43
510.44
447.84
418.43
: w-26.1
: BRUKER EQUINOX55
: RES 4.0cm-1 SCANS 256
: 2011-7-7

(**F**)

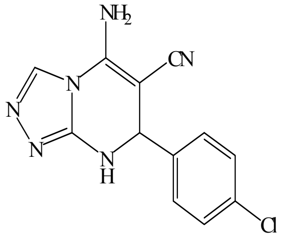
NH2
CN
N
N
N
N
H
Cl

4h

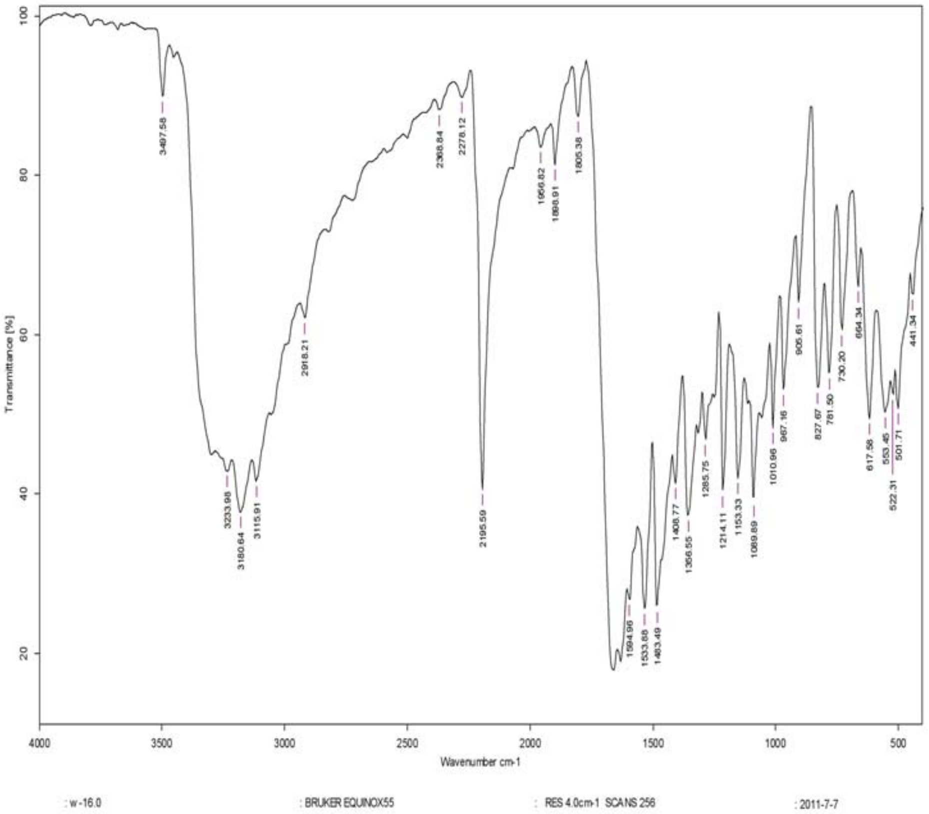
Transmittance [%]
Wavenumber cm-1
3497.58
3233.98
3180.64
3115.91
2918.21
2368.84
2278.12
2195.59
1956.82
1898.91
1805.38
1594.96
1533.88
1483.49
1408.77
1356.55
1285.75
1214.11
1153.33
1089.89
1010.96
967.16
905.61
827.67
781.50
730.20
664.34
617.58
553.45
522.31
501.71
441.34
: w -16.0
: BRUKER EQUINOX55
: RES 4.0cm-1 SCANS 256
: 2011-7-7

(**G**)

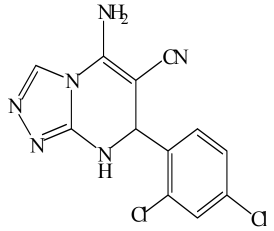
NH2
CN
N
N
N
N
H
Cl
Cl

4i

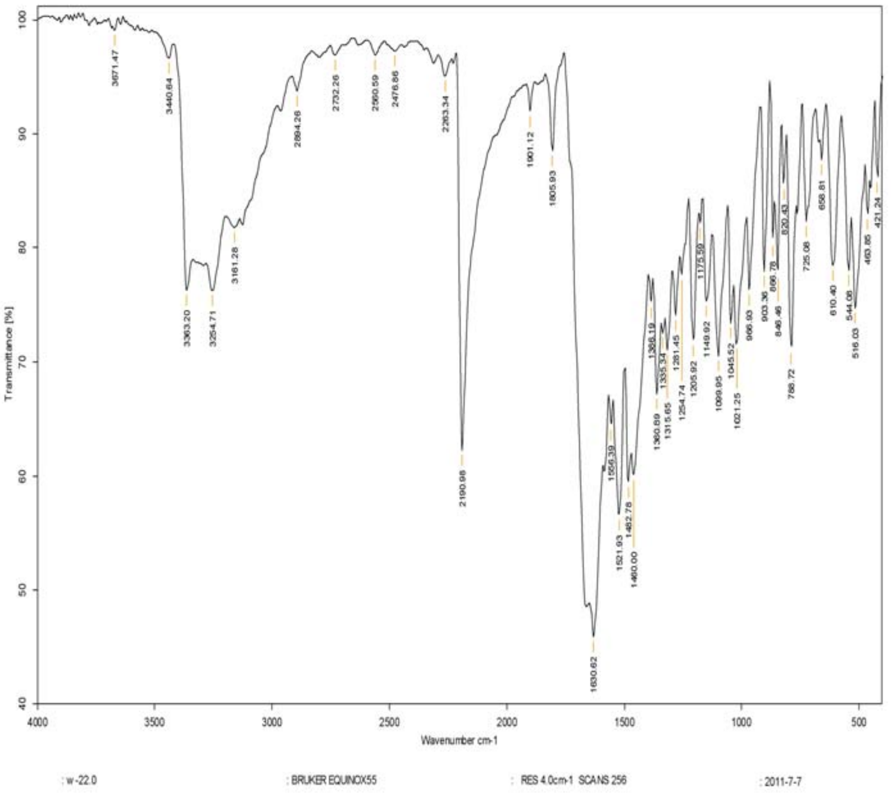
Transmittance [%]
Wavenumber cm-1
3671.47
3440.64
3363.20
3254.71
3161.28
2884.26
2732.26
2560.59
2476.86
2263.34
2190.98
1901.12
1805.93
1630.62
1566.39
1521.93
1482.78
1460.00
1388.19
1360.89
1335.34
1315.65
1281.45
1254.74
1205.92
1175.59
1149.92
1099.95
1045.52
1021.25
966.93
903.36
866.78
846.46
820.43
788.72
725.08
658.81
610.40
544.08
516.03
463.85
421.24
: w -22.0
: BRUKER EQUINOX55
: RES 4.0cm-1 SCANS 256
: 2011-7-7

(**H**)

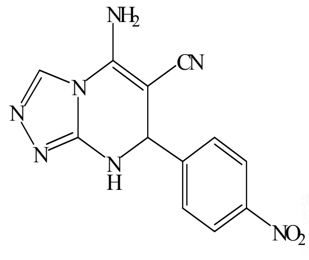
NH2
CN
N
N
N
N
H
NO2

4j

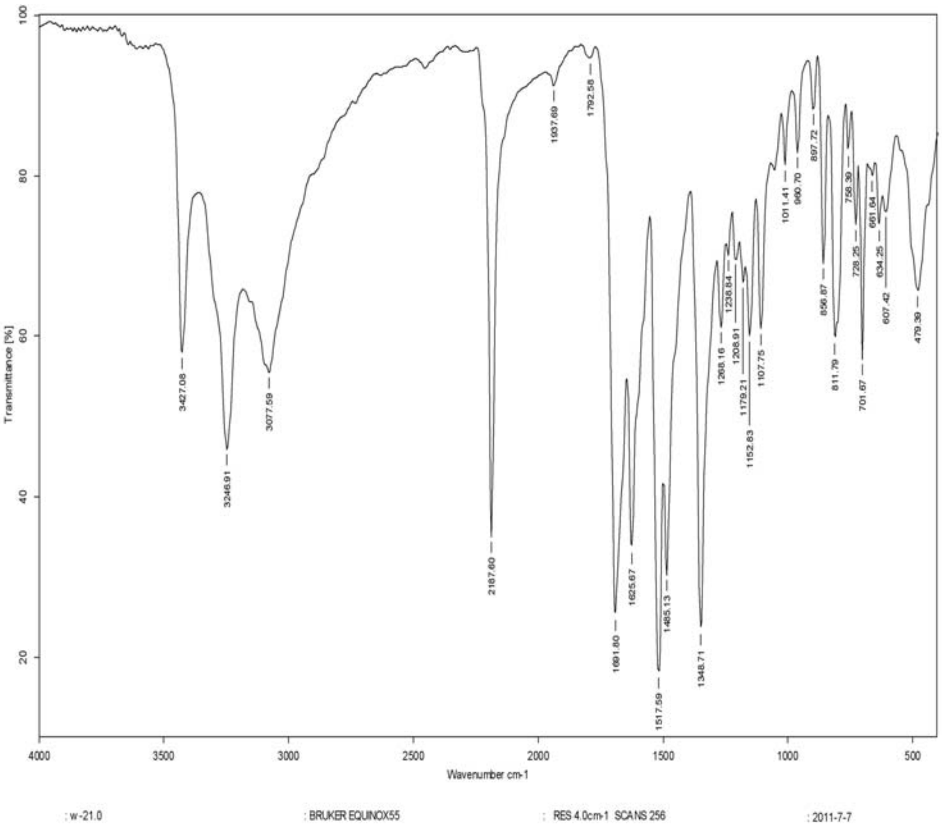
Transmittance [%]
Wavenumber cm-1
3427.08
3246.91
3077.59
2187.60
1937.69
1792.58
1691.80
1625.67
1517.59
1485.13
1348.71
1268.16
1238.64
1208.91
1179.21
1152.83
1107.75
1011.41
960.70
897.72
856.87
811.79
758.30
728.25
701.67
661.64
634.25
607.42
479.39
: w-21.0
: BRUKER EQUINOX55
: RES 4.0cm-1 SCANS 256
: 2011-7-7

(I)

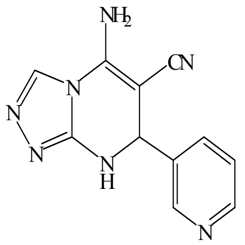
NH2
CN
N
N
N
H
N

4l

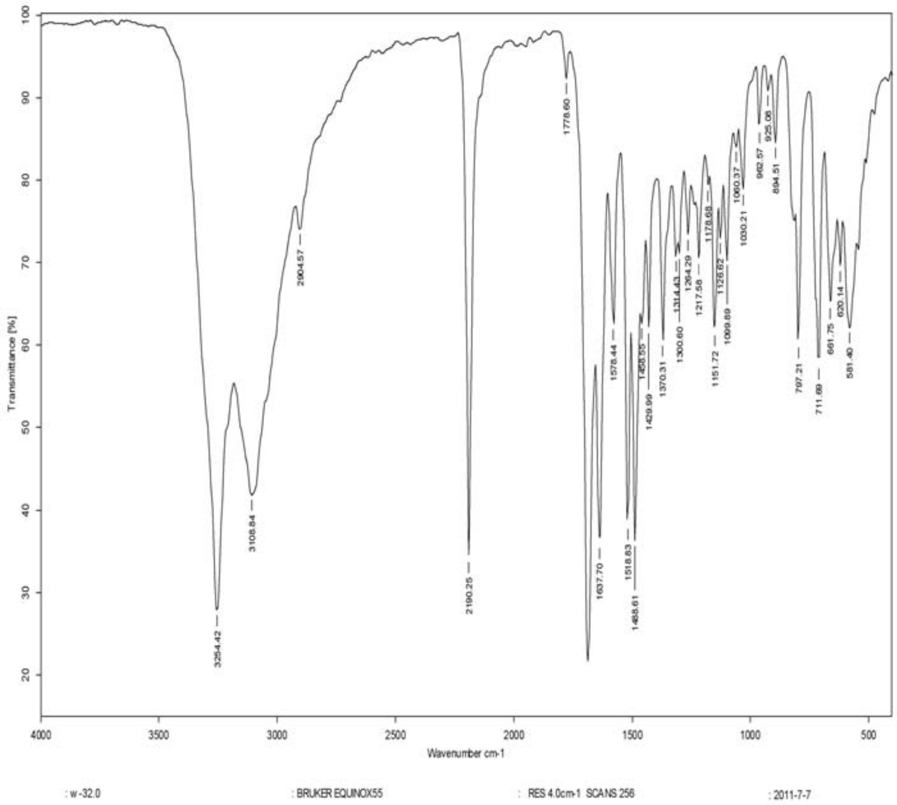
Transmittance [%]
Wavenumber cm-1
3254.42
3108.84
2904.57
2190.25
1778.60
1637.70
1578.44
1518.63
1488.61
1458.55
1429.99
1370.31
1314.43
1300.60
1264.29
1217.58
1178.65
1151.72
1128.62
1099.89
1060.37
1030.21
962.57
925.08
894.51
797.21
711.69
661.75
620.14
581.40
: w-32.0
: BRUKER EQUINOX55
: RES 4.0cm-1 SCANS 256
: 2011-7-7

(J)
